# Biomarkers in Renal Cell Carcinoma: A Systematic Review and Immunohistochemical Validation Study

**DOI:** 10.3390/cancers17152588

**Published:** 2025-08-06

**Authors:** Brett Berezowski, Robert Boothe, Billy Chaplin, Sharon J. Del Vecchio, Zakariya Fares, Tyrone L. R. Humphries, Keng Lim Ng, Taylor Noonan, Hemamali Samaratunga, Aaron Urquhart, David A. Vesey, Simon T. Wood, Glenda C. Gobe, Robert J. Ellis

**Affiliations:** 1 Kidney Disease Research Collaborative, Princess Alexandra Hospital and The University of Queensland, Translational Research Institute, Brisbane, QLD 4102, Australia; 2 Faculty of Health, Medicine and Behavioural Sciences, The University of Queensland, Brisbane, QLD 4102, Australia; 3 Princess Alexandra Hospital, Metro South Health, Brisbane, QLD 4102, Australia; 4 Urology Department, Frimley Park Hospital, Frimley GU16 7JG, UK; 5 Aquesta Uropathology, Brisbane, QLD 4066, Australia; 6 Kidney Health Service, Royal Brisbane and Women’s Hospital, Brisbane, QLD 4006, Australia

**Keywords:** kidney cancer, renal cell carcinoma, biomarkers, aminopeptidase A, aminopeptidase N, gamma-glutamyl transferase, neuron-specific enolase

## Abstract

*Background and Objectives*: The worldwide incidence of renal cell carcinoma (RCC) rose by 22% between 2012 and 2022. In Australia, RCC accounted for 2.8% of all cancer diagnoses and contributing to 1.8% of cancer-related deaths. Identification of RCC biomarkers may aid in diagnosis and management. *Methods*: A systematic review of immunohistochemical markers of RCC studies published between 1990 and 2019 was undertaken to select candidate biomarkers of RCC. Immunohistochemical staining of 73 clear cell RCC tumors and paired normal tissue was undertaken using selected markers. Semi-quantitative and quantitative analysis of staining intensity between paired samples was undertaken to evaluate utility as potential biomarkers, using Chi-square tests and paired *t*-tests for analysis. As an exploratory analysis, staining intensity was also compared on clinical/demographic variables using linear and logistic regression. *Results*: There were 123 candidate biomarkers identified in 91 studies. Four candidate markers were selected for further investigation: aminopeptidase A (APA)/cluster of differentiation (CD)249, aminopeptidase N (APN)/CD13, gamma-glutamyl transferase (GGT), and neuron-specific enolase (NSE). APA, GGT, and APN all demonstrated reduced staining intensity in the tumor compared with normal tissue (*p* < 0.001 for all). NSE demonstrated a statistically significant increase in expression in tumor compared with normal tissue (*p* < 0.001), and this was more pronounced in patients aged >60 years (*p* = 0.038). *Conclusions*: The utility of APA, APN, and GGT as diagnostic biomarkers in clear cell RCC is limited. NSE may have some role as a biomarker for clear cell RCC, particularly among older patients; however, further investigation is required.

## Simple Summary

The incidence of kidney cancer is rising. The development of better and more targeted diagnostic and therapeutic techniques may help to improve the management of kidney cancer. This study aimed to identify potential biomarkers that could assist with this goal. Of four potential biomarkers, only one was found to have a potential role in diagnosis and management of the most common form of kidney cancer, clear cell renal cell carcinoma.

## 1. Introduction

Renal cell carcinoma (RCC) stands as a significant and growing burden on healthcare systems, both in Australia and globally. The worldwide incidence of RCC has increased substantially, with an estimated 22% rise between 2012 and 2022 [1,2]. This may be a consequence of increased surveillance imaging and better diagnostic techniques and access to healthcare, as well as changing population demographics with a higher prevalence of chronic diseases which predispose to RCC development related to environmental exposures and lifestyle related risk factors [2]. This translates to approximately 400,000 new cases and 175,000 deaths annually worldwide [2]. Within Australia, the impact of RCC is also considerable. In 2022, an estimated 4552 new cases were diagnosed, representing 2.8% of all cancer diagnoses and contributing to 1.8% of cancer-related deaths [3].

The presentation of RCC is often asymptomatic, often with no noticeable biochemical changes on routine investigations [4]. Detection and management of RCC in early stages is associated with substantially better oncological and survival outcomes [5]. However, incidental diagnosis of renal masses on routine imaging has increased over time [6], which may have led to overdiagnosis and overtreatment of RCC [7]. Novel imaging approaches targeting biomarkers have a potential role in mitigating this issue, where sensitive and specific markers can be used to differentiate aggressive from less aggressive kidney tumors, as well as potentially deliver targeted therapeutics. A recent example in RCC is carbonic anhydrase IX (CAIX), where PET-CT using zirconium radiotracer conjugated girentuximab (anti-CAIX monoclonal antibody) was found to be 85.5% sensitive and 87% specific for diagnosing clear cell (cc) RCC in a phase 3 trial [8].

The identification and validation RCC biomarkers to improve non-invasive diagnostic approaches would be of great value in the evolving landscape of monoclonal antibody use in clinical applications. The aim of this study was to identify potential candidate biomarkers for RCC through systematic search of the existing literature, and to subsequently evaluate their utility using immunohistochemistry in a cohort of patients with ccRCC.

## 2. Methods

### 2.1. Systematic Review Search Strategy

The MEDLINE (PubMed) and EMBASE databases were queried by combining terms related to kidney cancer, kidney tissue, immunohistochemistry, and biomarkers (Supplementary Materials). The final combinations were piloted and optimized using Boolean operators, in consultation with an experienced biomedical librarian. The electronic searches were conducted between 17 and 20 September 2019. There was no registered review protocol. Search strategy and reporting was performed in accordance with the PRISMA (Preferred Reporting Items for Systematic Reviews and Meta-Analyses) guidelines [9].

### 2.2. Study Inclusion and Exclusion Criteria

Original research articles and conference abstracts that involved immunohistochemical analysis of human kidney tumor tissue and were published after 1 January 1990 were screened for inclusion. Studies were excluded if they utilized only cell culture models, were predominantly focused on familial cancer syndromes, recurrent RCC or metastatic tumors, included only patients with kidney failure/on dialysis or kidney transplant recipients, or had a small sample size (<30 participants).

Articles that did not address the key aims of this study were also excluded. Studies that utilized historical or outdated classification schemas for RCC were considered for exclusion on a case-by-case basis. Studies were also excluded if results were unable to be disaggregated for the purposes of quantitative assessment. Criteria for inclusion and exclusion were specified a priori. The PRISMA diagram is reported in Supplementary Figure S1.

### 2.3. Study Selection and Data Extraction

Title/abstract screening was undertaken by two independent reviewers (T.N., Z.F.). Results were compared, and discrepancies were resolved by a third reviewer (R.J.E.). Full-text screening, quality assessment, and data extraction was undertaken by three independent reviewers (B.C., R.B., R.J.E.).

### 2.4. Quality Assessment

Studies were assessed against eight criteria relating to methodological and technical accuracy, and were assigned a score of 0–8, where each criterion equates to one point: (i) clear evidence-based rationale for including selected protein biomarker(s); (ii) staining intensity was assessed quantitatively or semi-quantitatively; (iii) anatomical staining patterns were described; (iv) immunohistochemical methods were clearly described; (v) sample collection methods were clearly described; (vi) case sampling strategy was clearly described and rationalized; (vii) matched non-neoplastic controls were used; (viii) orthogonal methods were used to confirm results.

### 2.5. Quantitative Analysis

Studies were included in quantitative analysis if results indicating positive and negative (or negligible/weak) staining were broken down by case, such that staining across multiple studies could be combined to provide a percentage of cases that demonstrated positive staining for a particular protein. Results were separated into major histological subtypes (ccRCC, papillary RCC, chromophobe RCC, and renal oncocytoma). Results were also presented as the percentage of malignant tumors that stained positive, including other less common subtypes of RCC in this category, as well as some studies which did not report a breakdown of cases by histological subtype. Some studies reported staining in non-neoplastic cortical tissue, which was also presented in this analysis.

### 2.6. Immunohistochemistry Validation Cohort

Paired tumor and normal kidney tissue from a cohort of 73 patients who underwent nephrectomy for ccRCC was used to investigate the candidate biomarkers further [10]. Clinical variables were collected prospectively at the time of surgery through chart review, with retrospective re-review to minimize missing data. Age, sex, and comorbidities were obtained from medical records, while tumor characteristics were obtained from imaging and pathology reports. Human research ethics approval was obtained from Metro South Health (HREC/16/QPAH/353). All patients provided written informed consent for tissue collection. This study was conducted in accordance with the Declaration of Helsinki.

### 2.7. Tissue Preparation

Tissue collection and preparation has been described previously [11]. After nephrectomy, tissue was placed in 4% formalin for 24 h prior to being embedded in paraffin using a Leica EG1150 Tissue Embedder (Leica, Wetzlar, Germany). A TMA Grandmaster Automated Tissue Microarrayer (3DHISTECH, Budapest, Hungary) was used to develop a tissue microarray (TMA) using 0.6 mm cores. A Leica RM2245 Semi-Motorized Rotary Microtome (Leica, Wetzlar, Germany) was used to cut 4 µm sections.

### 2.8. Immunohistochemistry

From the systematic review, four candidate biomarkers were selected for further investigation with immunohistochemistry. Selection was based on a high percentage of strong positive staining in ccRCC (>95%), predominant expression at the cell membrane, and low membrane expression reported in normal kidney tissue. The candidate biomarkers were aminopeptidase A (APA)/cluster of differentiation (CD)249, aminopeptidase N (APN)/CD13, gamma-glutamyl transferase (GGT), and neuron-specific enolase (NSE). Antibodies were purchased from ThermoFisher Scientific (Waltham, MA, USA) and optimized using kidney tissue and a pan-cancer panel. The following dilutions were selected: APA/CD249 1:100; GGT1 1:500; NSE 1:100; APN/CD13 1:200. Immunohistochemistry was performed using a Ventana Discovery Ultra Automated Immunohistochemistry Platform (Roche Diagnostics Corporation, Indianapolis, IN, USA). See the Supplementary Materials for further details.

### 2.9. Image Analysis

TMA slides were imaged using an Olympus VS120 Brightfield Slide Scanner. QuPath image software was used for image analysis. Staining localization was manually identified for each sample in the control and RCC groups. The overall level of staining in each sample was graded as either strong, weak, or none. Examples of the staining levels are shown in Supplementary Figure S2. Change in staining in matched control and RCC tissue samples was evaluated and categorized. Samples that maintained their staining category were marked as no change (0). Samples with increased or decreased staining by 1 group (no stain to weak stain, or weak stain to strong stain) were marked as a small increase (+) and the opposite (strong stain to weak stain, or weak stain to no stain) were marked as a small decrease (−). Samples with increased staining of 2 groups (no stain to strong stain) were marked as a large increase (++) and those with decreased staining (strong stain to no stain) were marked as a large decrease (−−). Quantification of total staining values was measured by QuPath Pixel Classifier, which was manually trained to identify staining patterns for hematoxylin stain, positive antibody stain, and no staining. The pixel classifier measured the identified stains and quantified the total area (µm^2^) for each.

### 2.10. Analysis of Publicly Available Gene Expression Data

Analysis of publicly available gene expression data for the selected candidate biomarkers was undertaken to support the results of our immunohistochemical analysis. Data were obtained from the UCSC Xena Functional Genomics Explorer (https://xenabrowser.net/, accessed 27 August 2025). RCC tissue expression profiles were obtained from the TCGA Kidney Clear Cell Carcinoma (KIRC) cohort (*n* = 606). For control samples, normal healthy tissue expression data was sources from the harmonized TCGA TARGET GTEx dataset (*n* = 19,120). For both cohorts, gene expression values were provided as log2(normalized count + 1) transformed values, derived from RNA-Seq by expectation maximization counts. The UCSC Xena platform was used to undertake comparative analysis.

### 2.11. Statistical Analysis

GraphPad Prism 10.0.2 was used for statistical analysis and figure creation. Chi-square analysis was employed to compare staining intensities from the qualitative analysis. Fold change in total positive staining for each biomarker was calculated as the intensity of protein detection in the tumor tissue divided by the non-cancer tissue. Paired *t*-tests were used for quantitative analysis to compare total positive staining between control and RCC tissues for each biomarker. An exploratory analysis was undertaken to evaluate the relationships between clinical variables and staining intensity, using simple linear and logistic regression. Significant relationships were investigated further to evaluate whether clinical variables modified staining patterns. To analyze the effect of age and diabetes on biomarker detection, a Mann–Whitney U-test was utilized. A Mann–Whitney test was also used to compare mRNA expression data.

## 3. Results

### 3.1. Systematic Review

There were 91 studies (Supplementary Materials) included in the systematic review, evaluating 123 protein biomarkers. Thirty of these biomarkers were evaluated in two or more studies, and five biomarkers were evaluated in five or more studies. Most studies were of reasonably high methodological quality, with 64% (58/90) of included studies having a quality score of 5/8 or higher. The quantitative assessment of the percentage of cases that stained positive for each specific biomarker grouped by kidney tumor histological subtype is presented in Table 1.

APN/CD13, APA/CD249, GGT, and NSE were selected for in-depth analysis due to their promising differential expression in RCC versus control tissues. Specifically, APN was detected in 97% of RCC samples (33/34). Similarly, APA showed a 97% presence in RCC (33/34). GGT exhibited the highest sensitivity, being present in 100% of RCC samples (34/34). NSE was observed in 97% of RCC tissues (33/34).

### 3.2. Cohort Demographics

There were 73 patients with ccRCC included in the TMA. The mean age was 58 years old (range 32–84) and 59% were male. More extensive patient demographics are reported in Supplementary Table S1.

### 3.3. Immunohistochemistry

The TMA consists of 73 control and 73 RCC samples from matched patient donors. All 73 samples in the control group were found to have normal architecture. Some normal nephron architecture (tubules/glomeruli) was preserved in three (4.1%) tumor samples.

All tumor and non-tumor control samples stained positive for APN, APA, and GGT. In the control samples, staining was localized to the proximal tubules and glomerular basement membrane. In the tumor samples, APN, APA, and GGT displayed variable localization; however, in the three samples where normal structures were present, the staining location in these structures was comparable to the non-tumor controls. All control and RCC samples showed positive staining in the cytoplasm and cell membrane.

NSE staining was found in 16/73 (21.9%) of non-tumor control samples and 57/73 (78.1%) of tumor samples. There was variable localization of staining in both control and tumor samples, with expression throughout the cell membranes, nuclei and cytoplasm in normal tubular structures of non-cancer samples, and within the neoplastic cells.

### 3.4. Semi-Quantitative Image Analysis

Staining for each biomarker was classified as strong, weak, or absent staining for matched tumor and non-tumor tissue. Each matched sample was then evaluated for the change in staining category from the control to the RCC samples. Representative images are presented in Supplementary Figure S1. Results are reported in Table 2 and Supplementary Table S2.

The control group for APA showed strong staining in 89.0% of samples and weak staining in the remaining 11.0%. The APA RCC group had an overall reduced level of staining compared to the control. There was a significant difference in staining intensity groups when control was compared to RCC, with overall decreased staining intensity in the tumor tissue (*p* < 0.001). There was a large decrease in staining intensity groups between control and RCC in 6/73 (8.2%) samples, a small decrease in 49/73 (67.1%) samples, and no difference in 18/73 (24.7%) samples.

The control group for APN showed strong staining in all samples. The RCC group for APN had an overall reduced level of staining, with only 47.9% of samples staining strongly. There was a significant difference in staining intensity groups when control was compared to RCC, with overall decreased staining intensity in the tumor tissue (*p* < 0.001). There was a large decrease in staining intensity between the control and RCC in 11/73 (15.1%) samples, a small decrease in 27/73 (37.0%) samples, and no difference in 35/73 (47.9%) samples.

The control group for GGT showed strong staining in 94.5% of samples and weak staining in 5.5%. In the RCC tissue samples, only 11.0% had strong staining. There was a significant difference in staining intensity groups when the control was compared to RCC, with overall decreased staining intensity in the tumor tissue (*p* < 0.001). There was a large decrease in staining intensity between control and RCC in 12/73 (16.4%) samples, a small decrease in 50/73 (68.5%) samples, and no difference in 11/73 (15.1%) samples.

The control group for NSE showed weak staining in 78.1% samples and no staining in 21.9% samples. In the RCC group, strong staining was found in 42.5% of samples, whereas weak and no staining was found in 45.2% and 12.3%, respectively. There was a significant difference in staining intensities when controls were compared to RCC tissue samples, with an overall increase in staining intensity in the tumor tissue (*p* < 0.001). There was a large increase in staining intensity between control and RCC in 5/73 (6.8%) samples, a small increase in 31/73 (46.6%) samples, a small decrease in 3/73 (4.1%) samples, and no difference in 34/73 (46.6%) samples.

### 3.5. Quantitative Image Analysis

**Figure 1:**
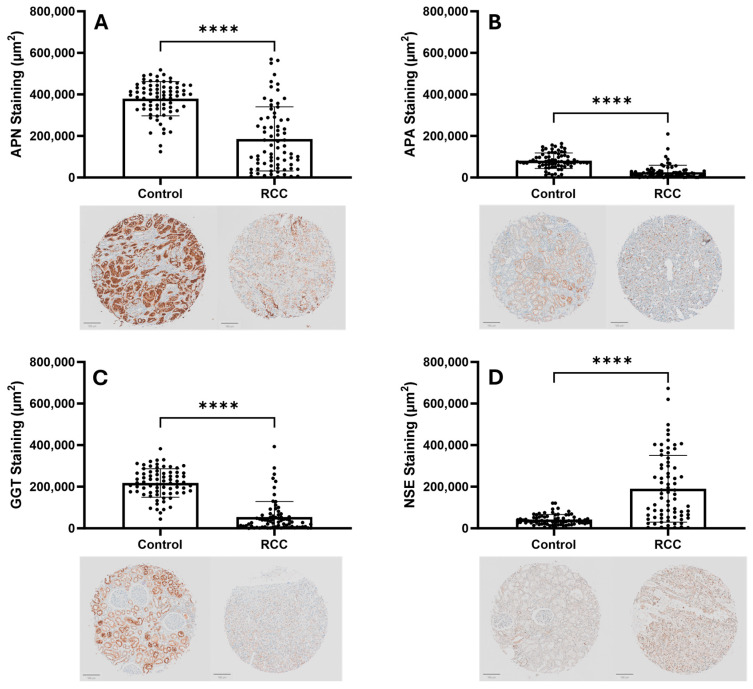
Comparison of total positive staining in control vs. clear cell RCC tissue samples for APN, APA, GGT, and NSE (*n* = 73). Total area of positive staining was measured and quantified using QuPath pixel classifier. Positive staining was found to be significantly elevated (*p* < 0.0001) over RCC tissue samples in APN (panel **A**), APA (panel **B**), and GGT (panel **C**). NSE staining was significantly elevated (*p* < 0.0001) in RCC tissues compared to control (panel **D**). Scale bar = 100 µm. Statistical analysis was performed with GraphPad Prism using paired *t*-tests. **** *p* < 0.0001.

Total positive staining area for APN, APA, and GGT was found to be significantly decreased (*p* < 0.0001) in RCC tissues compared to the matched control samples (Figure 1). There was a significant increase (*p* < 0.0001) in total positive staining area for NSE in RCC tissues compared to matched control samples (Figure 1).

### 3.6. Exploratory Correlations with Clinical Data

The relationship between clinical variables and staining intensity for each biomarker is presented in Supplementary Tables S3 and S4.

**Figure 2:**
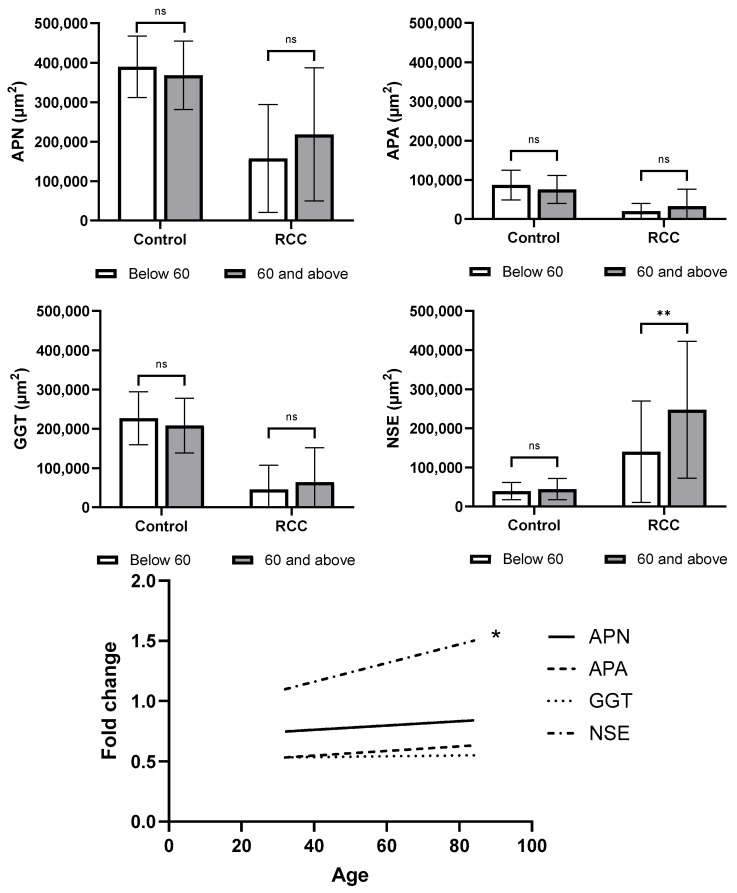
Comparison of biomarker detection above and below the age of 60 (*n* = 73). Samples were grouped based on age (above or below 60) and total positive staining for each biomarker was compared for both control and clear cell RCC tissues. APN, APA, and GGT showed no significant difference in positive staining levels in RCC or control (*p* > 0.05). NSE showed significantly elevated positive staining in RCC tissues for those aged over 60 compared to those under 60 (*p* = 0.008). Comparison was performed on GraphPad Prism with Mann–Whitney tests. ns indicates *p* > 0.05, * indicates *p* < 0.05, ** indicates *p* < 0.01. The fold change values were calculated to determine the overall change in biomarker detection in RCC tissues from control. These values were plotted against the age of the patient. Fold change values for APN, APA, and GGT biomarkers did not significantly correlate with age (*p* > 0.05). NSE fold change had a significant positive correlation with increasing age (*p* = 0.038). Statistical analysis was performed on GraphPad Prism using simple linear regression. * indicates *p* < 0.05.

When comparing biomarker expression by age (stratified as <60 and ≥60 years), there was a significant increase in the expression of NSE total area staining in tumor samples in the older group (*p* = 0.008). The difference between control and tumor staining was also significantly increased (*p* = 0.038). There was no difference in NSE staining in non-tumor tissue by age. There was no significant relationship between age and staining of APN, APA, or GGT in either control or tumor tissue (Figure 2).

When comparing biomarker expression by diabetes status, APA staining was significantly higher (*p* = 0.030) in control tissues but significantly lower (*p* = 0.049) in RCC tissues in non-diabetic patients compared to patients with diabetes. GGT staining showed no significant difference in control tissues but was significantly lower (*p* = 0.038) in the tumor tissue of non-diabetic patients. APN and NSE detection did not significantly differ between patients with diabetes and non-diabetic patients (Supplementary Figure S3).

There was no significant difference in staining intensity observed between T stage or nucleolar grade.

### 3.7. Gene Expression Analysis

**Figure 3:**
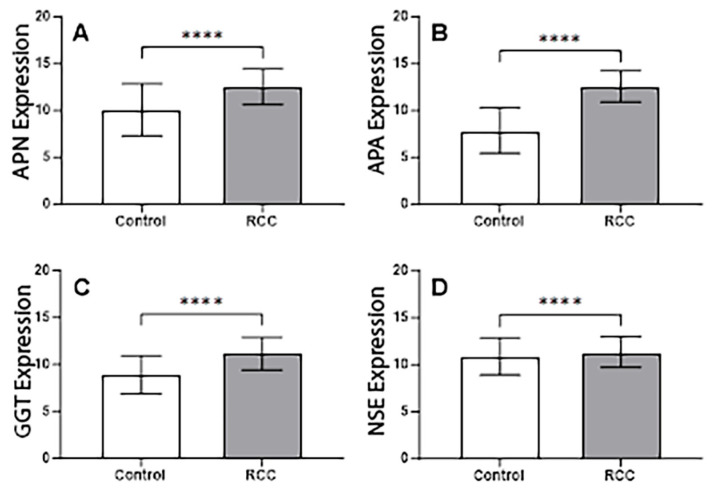
mRNA expression levels of APN (panel **A**), APA (panel **B**), GGT (panel **C**), and NSE (panel **D**) are compared between control (normal healthy tissues from TCGA TARGET GTEx) and RCC tissues (TCGA kidney clear cell carcinoma). Gene expression is quantified as log2(normalized count + 1) transformed values derived from RSEM counts. Data are presented as mean ± standard deviation. Statistical significance between control and RCC groups. **** *p*-value < 0.0001.

Analysis of publicly available mRNA expression data revealed significant differences in the expression levels of all four genes of interest between RCC tissues and normal control tissues. The genes for APN, APA, GGT, and NSE all exhibited significantly (*p* < 0.0001) higher expression in RCC tumor samples compared to their expression in healthy control tissues. These findings indicate an upregulation of these genes in the context of RCC (Figure 3).

## 4. Discussion

This study utilized a systematic review to identify four candidate biomarkers (APA, APN, GGT, and NSE) which may be useful in the clinical management of RCC, and subsequently evaluated the immunohistochemical staining patterns of these biomarkers in a diverse cohort of 73 patients managed surgically for ccRCC. Based on our results, APA, APN, and GGT were unlikely to have significant utility as a diagnostic biomarker in ccRCC, whereas NSE may have a limited role.

**Figure 4:**
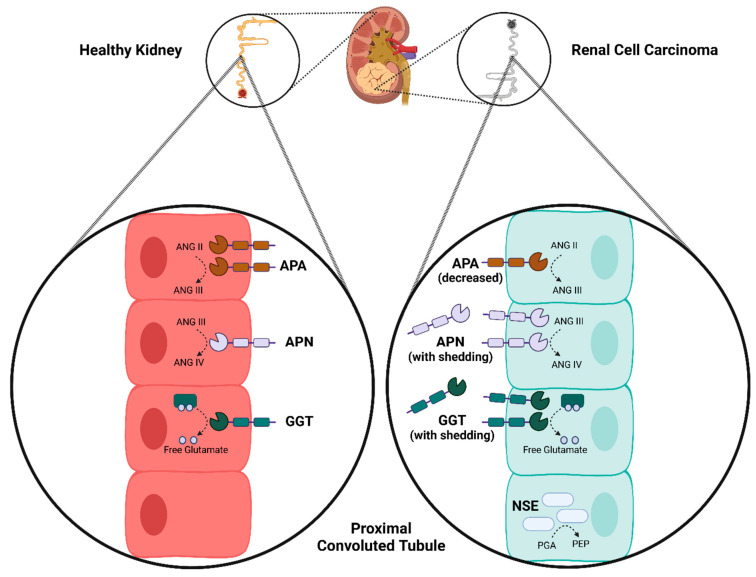
Localization and enzymatic activities of APN, APA, GGT, and NSE in healthy and RCC tissue. This figure compares the localization and basic enzymatic activities of alanyl aminopeptidase N (APN), aminopeptidase A (APA), gamma-glutamyl transferase (GGT), and neuron-specific enolase (NSE) in healthy renal tubule (**left**) and renal cell carcinoma (RCC) tissue (**right**). APN is membrane-bound in healthy tissue and increased and shed in RCC, with arrows indicating angiotensin III/IV processing. APA is membrane-bound in healthy tissue and shows variable expression in RCC, with arrows indicating angiotensin II/III processing. GGT is membrane-bound in healthy tissue and increased in RCC, with arrows indicating glutamate release. NSE is absent in healthy kidney tissue but present in RCC, with an inset showing neuron expression and arrows indicating PGA/PEP interconversion. Figure created with BioRender image software (https://www.biorender.com/) (2025).

APA expression has been previously documented within the cell membrane of the proximal renal tubules. Investigations utilizing mouse tissue samples identified APA within the proximal renal tubules and RCC [12]. In human choriocarcinoma cell lines, elevated APA levels were found specifically in the cytotrophoblast layer, as opposed to the syncytiotrophoblast of the placenta [13]. Both of these studies consistently localized APA to the cell membrane (Figure 4). Functionally, APA plays a role in the renin–angiotensin–aldosterone system (RAAS), contributing to blood pressure regulation. Its enzymatic activity involves the cleavage of the N-terminal aspartate from angiotensin II to generate angiotensin III, thereby impacting the RAAS cascade [14]. Due to its involvement in hypertension, APA has been explored as a potential target for novel antihypertensive agents. However, the expression and activity of APA within the context of RCC remain incompletely understood. A previous study reported a decrease in APA expression or activity in approximately 20% of surveyed RCC tissues; however, the underlying mechanism for this finding was not elucidated, and the study was limited by a small sample size [15]. Notably, in other malignancies, such as choriocarcinoma, elevated APA levels were found across multiple cell lines, suggesting its potential utility in malignancy detection when other markers are inconclusive [13]. For this reason, APA was selected in this study for further evaluation.

In the present study, APA was consistently localized to the proximal renal tubules and glomerular basement membrane in normal kidney tissue (strong staining in 89% and weak staining in 11%). In ccRCC tissues, there was no consistent pattern of localization when positive staining was present, and only 15.1% showed strong staining, while the remainder showed either weak or no detectable expression. At an individual sample level, a noticeable reduction in APA staining intensity was identified in 67% of the RCC samples, whereas 24.7% showed no appreciable difference. This pattern was clearly reflected in our qualitative assessment, which demonstrated a significant reduction in APA staining detection within the RCC tissues compared to the controls. The observed trend of decreased expression in the tumor samples coupled with the high degree of expression in both ccRCC and control tissues suggests that its utility in this specific diagnostic context is limited.

APN has been identified in both healthy and malignant renal tissue samples. While primarily localized to the cell membrane, soluble forms have also been detected. In healthy tissue, antibodies specific for APN have shown positive staining on the cell membranes of osteoclasts, fibroblasts, and endothelial cells lining the proximal renal tubules and bile duct canaliculi [16]. Furthermore, the measurement of soluble APN was found to be elevated in malignant effusions and intratumoral fluid of RCC tissues [17], suggesting potential shedding by tumors or the tumor vasculature in the kidney (Figure 3). Functionally, APN is described as playing a role in signal transduction, endocytosis, angiogenesis, and cell adhesion, with its enzymatic activity involved in the processing of angiotensin III and IV peptide hormones [18]. Given these diverse roles, APN has been highlighted as a potentially important target for cancer therapies and a biomarker for RCC. The role of APN in cancer tissues has not been fully defined; however, the inhibition of APN has been shown to significantly decrease angiogenesis and suppress tumor growth in mammary fat pad tumors [19]. Altogether, it appears that APN has a role in angiogenesis during tumor growth, which may be a potential therapeutic target.

In our cohort, APN was consistently localized to the proximal renal tubules and glomerular basement membrane of normal kidney tissue with strong staining present in all samples. There were variable localization patterns in ccRCC, and strong staining in only 47.9% of samples, with overall reduced expression of APN in ccRCC compared with matched normal tissue. While APN is clearly present in both ccRCC and normal kidney tissue, its reduced expression in the tumor samples suggests that its utility as a diagnostic biomarker for RCC is limited.

Expression of GGT has been found as a cell-surface-bound protein in kidney (Figure 3) and lung samples from both mouse and human cell lines [20]. The enzymatic action of GGT is to release free glutamate from various gamma-glutamyl compounds to aid in their metabolism [21]. These compounds can belong to groups such as antioxidants, drug metabolites, inflammatory mediators, and neuroactive compounds [22]. The breakdown of some drug metabolites in the kidney facilitated by GGT could convey resistance to chemotherapeutic drugs used in some cancers, such as RCC. Thus, it was proposed in 2009 that the use of GGT inhibitors may sensitize the kidney tissue to chemotherapy [23]. Since then, it has been shown that the inhibition of GGT increases the effectiveness of standard chemotherapies in RCC, and additionally, decreases cell migration and tumor growth through increased cell-cycle arrest [24]. GGT inhibitors are also under investigation for their potential benefits in other conditions such as cardiovascular disease, asthma, Parkinson’s disease, and other cancers [21,22]. Serum levels of GGT measured before nephrectomy has been shown to aid in prognosis and patient counselling [25]. The mechanism for poor prognosis with elevated GGT serum levels is due to higher oxidative stress in the tumor microenvironment and increased metastatic spread of disease [26]. Therefore, the use of GGT as a prognostic biomarker has been proposed, but its role in diagnosis is undetermined.

In our study, GGT was consistently localized to the proximal renal tubules and glomerular basement membrane of normal kidney tissue in 94.5% of cases. In ccRCC tissue, only 11% demonstrated strong staining. A decrease in staining intensity between normal and ccRCC tissue was seen in 84.9% of cases. Based on this, the utility of GGT as a diagnostic biomarker for ccRCC was limited.

Vertebrates express three isoforms of enolase, α, β, and γ, which all have different characteristic tissue distributions. Enolase α is ubiquitous, enolase β is muscle specific, and enolase γ is neuron specific (NSE) [27]. NSE is involved in both glycolysis and gluconeogenesis through opposite reactions. In glycolysis, NSE catalyzes the dehydration of 2-phospho-D-glycerate (PGA) to phosphoenolpyruvate (PEP), as well as the reverse reaction, the hydration of PEP to PGA in gluconeogenesis [27]. Immunostaining techniques have shown NSE in various neuron types including, but not limited to, Purkinje fibers, granule cells, and sensory and autonomic neurons [27]. Expression of NSE in other tissues, such as healthy kidney, has not been illustrated. However, NSE detection in RCC has been described through immunohistochemistry of tissue microarrays [28]. The finding of NSE in RCC but not healthy kidney tissue is likely only possible due to the process of neuroendocrine differentiation [29]. Recently, a study examining the risk factors for intraocular metastasis from RCC determined that increased levels of NSE were found in patients with intraocular metastases [30].

In our cohort, NSE exhibited non-specific staining in both control and RCC tissues, unlike the consistent localization of APN, APA, and GGT to the proximal tubules and glomerular basement membrane in control samples. Strong NSE staining was absent in all control tissues (0%), while a substantial proportion of RCC samples (42.5%) exhibited intense staining. Notwithstanding, a significant percentage of control tissues (78.1%) still displayed weak NSE staining. The analysis of individual patient-matched samples unveiled a high degree of variability in NSE expression. A considerable portion (46.6%) showed no change in staining intensity between normal and tumor tissue, and a small minority (4.1%) displayed a slight decrease. Conversely, a significant number showed a moderate increase (42.5%), and a few showed a large increase (6.8%) in NSE staining. Overall, quantitative analysis demonstrated a significant upregulation of NSE in RCC samples compared to controls. While this upregulation suggests a potential role as a diagnostic biomarker, the observed variability at the individual level and the presence of NSE in control tissues present significant challenges. The inconsistent expression pattern, where some RCC samples exhibit intense staining while others show no change, limits its reliability as a consistent diagnostic marker. A potential explanation for this variability may be attributed to patient demographics. Specifically, a positive correlation between NSE expression and increasing age was observed, particularly within RCC tissues, suggesting that NSE may be more useful in older patient populations. However, no other patient factors were found to significantly influence NSE detection. This age-dependent increase, while notable, does not mitigate the fundamental issue of variability. Furthermore, differences in staining patterns with significant degree of intracellular staining may limit its usefulness as a target for antibody-conjugated radiotracers. Nonetheless, further investigation into the utility of NSE as a diagnostic biomarker is reasonable, with a potential focus on older age groups.

There were some statistically significant differences in the expression of APA and GGT between patients with and without diabetes. The discordant results between tumor and non-tumor tissue for APA (lower in non-tumor compared with higher in tumor for diabetics), as well as the small effect size, raise potential that this represents a type I statistical error. Serum GGT expression has been reported to be higher in patients with diabetes potentially due to differences in lipid accumulation and inflammation [31]. While higher GGT expression was seen in the ccRCC samples, this pattern was not present in the nonneoplastic kidney tissue. The biological significance of this result is also uncertain.

The findings from the publicly available mRNA expression data, consistently indicate an upregulation of APN, APA, GGT, and NSE at the level of transcription in RCC tissues compared to healthy controls. However, our complementary protein staining experiments present a more complex picture, revealing reduced protein levels for APN, APA, and GGT in RCC tissues, while NSE protein staining aligns with the mRNA data by showing increased levels. This discordance between mRNA and protein expression for APN, APA, and GGT suggests potential post-transcriptional or post-translational regulatory mechanisms at play in RCC. Hypotheses for these differences include altered mRNA stability, translational repression by microRNAs or RNA-binding proteins, increased protein degradation rates, or differential protein secretion/localization in the tumor microenvironment that affects detection by immunohistochemistry.

Strengths of this study include a systematic approach to candidate biomarker selection and a modestly large sample size of ccRCC and matched normal tissue to explore the utility of these biomarkers. In particular, this approach overcame the risk of publication bias that would have arisen from using the pooled results from the systematic review without subsequently demonstrating reproducibility in an independent patient cohort. Due to the nature of the multifaceted approach of this study and delays that arose due to the COVID-19 pandemic, a limitation is that the systematic review results are outdated, and potential biomarkers reported after 2019 are not included. Notwithstanding, the results of the review reflect the information available to investigators at the time of biomarker selection. Given that the focus of the study was on patients who underwent surgical treatment, it is possible that the more advanced ccRCC may have different biomarker profiles than those included in this study. We also did not investigate biomarker expression in other RCC subtypes, which is a further limitation.

## 5. Conclusions

Overall, our study suggests that APA, APN, and GGT have no significant role as diagnostic biomarkers in ccRCC, while NSE may have a limited role. APN and APA exhibited a strong decrease in expression in RCC compared to control tissues. GGT, despite its known involvement in tumor progression, also presented with reduced expression in RCC samples. NSE, while showing overall upregulation in RCC tissues, was characterized by significant inter-individual variability and detectable expression in some control samples, which limits its diagnostic application and reliability. The variability of NSE expression, particularly its observed correlation with increasing patient age, further demonstrates the need for cautious interpretation, but also opens avenues for further investigation.

## Figures and Tables

**Table 1:** Potential biomarkers for RCC identified in the systematic review.

Biomarker	Studies (n)	Quality Score (Range)	ccRCC (n, %)	pRCC (n, %)	chRCC (n, %)	RO (n, %)	Malignant (n, %)
11β-HSD2	1	5	2/75 (2.6)	0/14 (0)	19/20 (95)	14/14 (100)	21/109 (19)
AEG-1	1	5	41/86 (47)	-	-	-	53/102 (52)
AMACR	3	3–5	10/11 (91)	10/10 (100)	4/11 (36)	19/33 (58)	36/80 (45)
Aminopeptidase A/CD249	1	3	33/34 (97)	-	1/4 (25)	0/4 (0)	35/40 (88)
AMY-1a	1	5	0/60 (0)	0/40 (0)	7/54 (13)	75/75 (100)	7/154 (4.5)
Arrestin-1	1	6	8/17 (47)	4/6 (67)	5/6 (83)	9/10 (90)	17/29 (59)
BAP-1	1	4	169/187 (90)	61/61 (100)	17/17 (100)	34/34 (100)	247/265 (93)
Bax	1	4	-	-	10/10 (100)	10/10 (100)	10/10 (100)
β-catenin	2	5	40/41 (98)	-	1/1 (100)	-	55/56 (98)
Bcl-2	1	4	-	-	28/28 (100)	33/33 (100)	28/28 (100)
CA-IX	3	3–5	61/67 (91)	8/10 (80)	1/3 (33)	23/42 (55)	123/141 (87)
Cathepsin	1	6	43/150 (29)	-	-	-	43/150 (29)
Cav-1	2	4	29/32 (91)	1/20 (5.0)	20/36 (56)	1/27 (3.7)	61/103 (59)
CD3	2	4	19/19 (100)	-	3/3 (100)	-	49/49 (100)
CD4	2	4	16/17 (94)	-	3/3 (100)	-	43/47 (91)
CD8	2	4	17/19 (89)	-	3/3 (100)	-	42/49 (86)
CD9	1	6	31/66 (47)	13/13 (100)	5/5 (100)	2/2 (100)	50/88 (57)
CD10	5	1–4	60/80 (75)	16/27 (59)	10/50 (20)	9/56 (16)	95/171 (56)
CD11	2	4–5	19/19 (100)	-	3/3 (100)	-	43/57 (75)
CD13/APN	1	5	33/34 (97)	-	0/4 (0)	0/4 (0)	34/40 (85)
CD14	1	4	19/19 (100)	-	3/3 (100)	-	48/48 (100)
CD15/LeuM-1	1	3	-	-	1/21 (4.8)	-	1/21 (4.8)
CD26/DP-4	1	5	34/34 (100)	2/4 (50)	4/4 (100)	1/2 (50)	40/42 (95)
CD31/PECAM-1	2	4	50/50 (100)	8/8 (100)	5/5 (100)	-	67/67 (100)
CD44-6v	1	5	14/68 (21)	-	6/11 (55)	0/8 (0)	23/92 (25)
CD44-9v	1	5	8/68 (12)	-	10/11 (91)	0/8 (0)	20/92 (22)
CD44s	1	5	22/68 (32)	-	10/11 (91)	0/8 (0)	36/92 (39)
CD56/NCAM	3	4–6	26/63 (41)	10/21 (48)	3/8 (38)	9/9 (100)	40/95 (42)
CD73	1	4	81/157 (52)	-	-	-	81/157 (52)
CD105	1	4	-	-	-	-	39/45 (87)
CD117/c-KIT	8	3–6	20/988 (2.0)	14/221 (6.3)	188/216 (87)	123/139 (88)	225/1470 (15)
CD147/EMMPRIN	1	5	313/322 (97)	36/42 (86)	22/22 (100)	8/8 (100)	371/386 (96)
Chromogranin A	1	6	0/34 (0)	0/9 (0)	0/2 (0)	-	0/45 (0)
CK (pan)	1	5	117/125 (94)	20/20 (100)	22/22 (100)	62/66 (94)	159/167 (95)
CK2a	1	6	80/105 (76)	18/27 (67)	7/8 (88)	12/13 (92)	107/142 (75)
CK7	11	1–6	38/287 (13)	56/76 (74)	97/119 (82)	80/176 (45)	236/532 (44)
CK8	3	1–5	40/156 (26)	24/37 (67)	44/64 (69)	45/74 (61)	109/257 (42)
CK8-18L	1	5	143/155 (92)	35/58 (92)	58/64 (91)	94/95 (99)	250/271 (92)
CK18	4	1–5	143/155 (92)	35/38 (92)	58/64 (91)	94/95 (99)	250/271 (92)
CK19	3	1–5	37/155 (24)	32/36 (89)	24/64 (38)	32/72 (44)	93/255 (36)
CK20	1	1	2/30 (6.7)	1/16 (6.3)	3/21 (14)	0/8 (0)	6/68 (8.8)
Cks	1	5	82/384 (21)	8/81 (9.9)	0/17 (0)	-	90/482 (19)
Claudin-7	1	4	0/33 (0)	4/19 (21)	4/6 (67)	2/6 (33)	8/58 (14)
Clusterin	1	6	-	-	-	-	65/67 (97)
Dcr-3	1	6	39/464 (8.4)	6/48 (13)	2/25 (8)	-	52/560 (9.3)
Desmin	1	5	-	-	-	-	4/30 (13)
DNMT-1	1	6	50/89 (56)	-	-	-	56/111 (50)
E-cadherin	3	3–5	18/154 (12)	3/20 (15)	15/56 (95)	16/16 (100)	77/233 (33)
EpCAM	1	5	131/318 (41)	-	-	-	131/318 (41)
EPO	1	6	33/34 (97)	0/9 (0)	0/2 (0)	-	33/45 (73)
ERa36	1	4	27/67 (40)	4/6 (67)	14/19 (74)	-	48/99 (48)
Erg	1	3	87/184 (47)	11/14 (79)	3/13 (23)	3/3 (100)	101/211 (48)
Estrogen Rec.	1	5	-	-	-	-	0/29 (0)
EZH2	1	6	334/422 (79)	48/55 (87)	12/23 (52)	-	411/520 (79)
Fas	1	5	18/20 (90)	11/11 (100)	17/20 (85)	-	46/51 (90)
FasL	1	5	20/20 (100)	11/11 (100)	20/20 (100)	-	51/51 (100)
FGFR-1	1	5	14/16 (88)	10/12 (83)	5/5 (100)	9/9 (100)	29/33 (88)
FGFR-2	1	6	-	49/214 (23)	-	-	49/214 (23)
FXYD-2	1	6	2/15 (13)	0/11 (0)	26/27 (96)	5/30 (17)	28/53 (53)
Galectin-1	1	8	87/91 (96)	-	-	-	170/182 (93)
GATA-3	2	2–4	1/116 (0.9)	0/53 (0)	2/33 (6.1)	9/47 (19)	6/241 (2.5)
GGT	1	5	34/34 (100)	-	0/4 (0)	1/4 (25)	35/40 (88)
GLTSCR-2	1	5	46/75 (61)	-	-	-	127/159 (80)
Glucocorticoid Receptor	1	6	97/147 (66)	6/23 (26)	1/17 (5.9)	2/14 (14)	104/187 (56)
GPC-3	1	4	27/502 (5.4)	16/62 (26)	32/40 (80)	12/21 (57)	75/604 (12)
HER-2	1	6	-	-	-	-	7/42 (17)
HLA-I	2	4–5	74/120 (62)	-	-	-	107/162 (66)
HLA-II	1	7	8/12 (67)	2/10 (20)	3/7 (43)	1/5 (20)	13/29 (45)
HLA-B,C	1	7	12/12 (100)	10/10 (100)	3/7 (43)	5/5 (100)	29/33 (88)
HLA-G	1	7	7/12 (58)	0/10 (0)	0/7 (0)	0/5 (0)	7/33 (21)
HMGB-1	1	5	-	-	-	-	71/80 (89)
Hsp-27	1	5	16/19 (84)	0/2 (0)	1/3 (33)	0/1 (0)	17/24 (71)
IFN-γ	1	6	16/60 (27)	-	-	-	16/60 (27)
KL-1	1	2	-	-	15/21 (71)	86/103 (83)	15/21 (71)
Ksp-cadherin	2	5–6	20/161 (12)	13/71 (18)	18/67 (27)	28/74 (28)	51/302 (17)
LRAT	1	4	13/13 (100)	7/7 (100)	6/6 (100)	4/4 (100)	27/27 (100)
Lysozyme	1	2	-	-	6/21 (29)	10/103 (9.7)	6/21 (29)
MAGE-A3/4	1	4	-	-	7/18 (39)	15/17 (88)	7/18 (39)
MIA	1	3	-	-	-	23/24 (96)	14/14 (100)
Mineralocorticoid Receptor	1	3	0/75 (0)	14/14 (100)	9/10 (90)	13/14 (93)	23/108 (21)
MOC-31	1	4	8/10 (80)	-	22/23 (96)	2/8 (25)	36/48 (75)
MSH-2	1	3	53/129 (41)	-	-	-	53/129 (41)
MUC-1	7	1–6	215/325 (66)	58/82 (71)	110/115 (96)	142/164 (87)	400/551 (73)
N-cadherin	2	2–3	8/19 (42)	-	2/37 (5.4)	0/16 (0)	10/56 (18)
NDUFA4L2	1	5	70/86 (81)	-	-	-	70/86 (81)
NEP	1	5	33/34 (97)	-	1/4 (25)	0/4 (0)	34/40 (85)
NPM	1	4	18/40 (45)	5/7 (71)	0/8 (0)	9/9 (100)	27/61 (44)
NSE	1	6	33/34 (97)	-	0/9 (0)	0/2 (0)	33/43 (77)
NY-ESO-1	1	4	-	-	6/18 (33)	15/17 (88)	6/18 (33)
OSCAR	1	3	-	-	-	22/22 (100)	13/13 (100)
p27Kip1	2	4–5	263/524 (50)	20/81 (25)	3/17 (18)	-	286/622 (46)
p53	3	3–7	17/52 (33)	5/10 (50)	24/39 (62)	30/42 (71)	46/101 (45)
PAX-2	1	6	29/30 (97)	17/30 (57)	10/30 (33)	27/30 (90)	56/90 (62)
PAX-8	2	2–4	89/104 (86)	19/21 (90)	9/11 (81)	20/21 (95)	124/145 (86)
Paxillin	1	4	1/65 (1.5)	1/14 (7.1)	6/6 (100)	2/2 (100)	9/89 (10)
PBMR-1	2	4–8	210/506 (42)	85/103 (83)	22/39 (56)	34/42 (81)	332/671 (49)
PD-L1	1	3	-	5/50 (10)	2/36 (5.6)	4/13 (31)	7/86 (8.1)
p-Glycoprotein	1	4	30/75 (40)	-	-	-	30/75 (40)
Progesterone Receptor	2	3–5	3/11 (27)	3/10 (30)	3/11 (27)	6/9 (67)	9/61 (15)
pSTAT-3	1	5	25/42 (60)	4/7 (57)	8/32 (25)	4/15 (27)	37/81 (46)
RAGE	1	5	-	-	-	-	72/80 (90)
RASSF1A	1	6	33/60 (55)	-	-	-	33/60 (55)
RECK	1	5	31/322 (9.6)	20/43 (47)	12/22 (55)	7/8 (88)	63/387 (16)
Recoverin	1	4	-	-	-	11/12 (92)	26/38 (68)
RET	1	5	0/18 (0)	34/66 (52)	4/10 (40)	4/4 (100)	49/107 (46)
RON	2	3–4	53/82 (65)	38/38 (100)	65/75 (87)	84/86 (98)	156/195 (80)
S100A1	2	3–5	30/41 (73)	34/36 (94)	6/61 (9.8)	41/44 (93)	79/147 (54)
SCF	1	5	40/40 (100)	25/25 (100)	19/19 (100)	27/27 (100)	84/84 (100)
Secretagogin	1	4	35/94 (37)	0/37 (0)	0/24 (0)	0/30 (0)	35/155 (23)
Skp-2	1	5	68/384 (18)	3/81 (3.7)	0/17 (0)	-	71/482 (15)
Synaptophysin	1	6	2/34 (5.9)	0/9 (0)	0/2 (0)	-	2/45 (4.4)
TFE-3	1	5	4/25 (16)	0/6 (0)	1/9 (11)	-	5/40 (12.5)
TFE-B	1	5	0/25 (0)	0/6 (0)	0/9 (0)	0/1 (0)	0/40 (0)
TFF-1 (pS2)	1	4	15/60 (25)	-	-	-	15/50 (25)
THP	1	3	-	-	0/21 (0)	-	0/21 (0)
TPI-1	1	5	18/19 (95)	1/2 (50)	0/3 (0)	0/1 (0)	19/24 (79)
uPA	1	6	3/17 (18)	-	-	-	3/18 (17)
uPAR	1	6	14/17 (82)	-	-	-	15/18 (83)
VEGF	1	4	25/31 (84)	6/8 (75)	1/2 (50)	-	34/44 (77)
VHL	1	8	77/317 (24)	13/40 (33)	4/22 (18)	4/8 (50)	102/400 (26)
Vimentin	5	1–6	96/132 (73)	52/62 (84)	3/102 (2.9)	39/174 (22)	167/313 (53)
VLA-4	1	4	19/19 (100)	-	3/3 (100)	-	23/23 (100)

Biomarkers identified in the systematic review of 91 studies investigating immunohistochemical staining in renal cell carcinoma (RCC). Grouped by clear cell (cc), papillary (p) and chromophobe (ch) RCC, benign renal oncocytoma (RO), and total malignant tumor (including less common subtypes). Due to the number of included biomarkers, abbreviations of the included proteins are reported in the Supplementary Materials.

**Table 2:** Immunohistochemistry semi-quantitative analysis.

	Control	ccRCC
Aminopeptidase A (APA)		
Strong	65 (89.0%)	11 (15.1%)
Weak	8 (11.0%)	55 (75.3%)
No Stain	0 (0%)	7 (9.6%)
Aminopeptidase N (APN)		
Strong	73 (100%)	35 (47.9%)
Weak	0 (0%)	27 (37.0%)
No Stain	0 (0%)	11 (15.1%)
Gamma-glutamyl transferase (GGT)		
Strong	69 (94.5%)	8 (11.0%)
Weak	4 (5.5%)	52 (71.2%)
No Stain	0 (0%)	13 (17.8%)
Neuron-specific enolase (NSE)		
Strong	0 (0%)	31 (42.5%)
Weak	57 (78.1%)	33 (45.2%)
No Stain	16 (21.9%)	9 (12.3%)

Abbreviations: ccRCC, clear cell renal cell carcinoma.

## Data Availability

Data related to the systematic review are contained within the article; additional data are available from the corresponding author upon request. Publicly available databases were used for gene expression analysis. The data source was cited within the body of the manuscript. Patient-level data reported in this article are not readily available due to individual privacy considerations.

## Supplementary Materials

The following supporting information can be downloaded at https://www.mdpi.com/article/10.3390/cancers17152588/s1. Figure S1: PRISMA diagram for systematic review; Figure S2: Example images for staining intensity categorization; Figure S3: Comparison of biomarker detection in diabetic and non-diabetic patients; Table S1: Cohort characteristics; Table S2: Change in staining intensity; Table S3: Correlation of total staining values for APN, APA, GGT, and NSE in control tissue samples with various parameters in the patient data; Table S4: Correlation of total staining values for APN, APA, GGT, and NSE in RCC tissue samples with various parameters in the patient data.
