# Correction: Berezowski et al. Biomarkers in Renal Cell Carcinoma: A Systematic Review and Immunohistochemical Validation Study. *Cancers* 2025, *17*, 2588

**DOI:** 10.3390/cancers18101549

**Published:** 2026-05-11

**Authors:** Brett Berezowski, Robert Boothe, Billy Chaplin, Sharon J. Del Vecchio, Zakariya Fares, Tyrone L. R. Humphries, Keng Lim Ng, Taylor Noonan, Hemamali Samaratunga, Aaron Urquhart, David A. Vesey, Simon T. Wood, Glenda C. Gobe, Robert J. Ellis

**Affiliations:** 1 Kidney Disease Research Collaborative, Princess Alexandra Hospital and The University of Queensland, Translational Research Institute, Brisbane, QLD 4102, Australia; 2 Faculty of Health, Medicine and Behavioural Sciences, The University of Queensland, Brisbane, QLD 4102, Australia; 3 Princess Alexandra Hospital, Metro South Health, Brisbane, QLD 4102, Australia; 4 Urology Department, Frimley Park Hospital, Frimley GU16 7JG, UK; 5 Aquesta Uropathology, Brisbane, QLD 4066, Australia; 6 Kidney Health Service, Royal Brisbane and Women’s Hospital, Brisbane, QLD 4006, Australia

## Text Correction

There was an error in the original publication [1]. The size of the tissue cores used to develop the tissue microarray was incorrectly reported as 1 mm instead of 0.6 mm. A correction has been made to Section 2.7. Tissue Preparation in Section 2. Methods.

A TMA Grandmaster Automated Tissue Microarrayer (3DHISTECH, Budapest, Hungary) was used to develop a tissue microarray (TMA) using 0.6 mm cores. A Leica RM2245 Semi-Motorized Rotary Microtome (Leica, Wetzlar, Germany) was used to cut 4 µm sections.

The authors apologize for any inconvenience caused and state that the scientific conclusions are unaffected. This correction was approved by the Academic Editor. The original publication has also been updated.
