# Supplementary material for: Biomarkers in Renal Cell Carcinoma: A Systematic Review and Immunohistochemical Validation Study
Source: Cancers (Basel). 2025 Aug 6;17(15):2588. doi: 10.3390/cancers17152588 (PMC12346209; doi:10.3390/cancers17152588)
Supplement: Supplementary file 1 [file cancers-17-02588-s001.zip › cancers-3762028-supplementary.pdf]

Supplementary Material

Systematic Review Search Terms..2
Antibodies used for immunohistochemistry 3
List of publications included in the systematic review..4
Biomarker abbreviations from Table 1 8
Supplementary Figures 11
Supplementary Tables...14

Systematic Review Search Terms

History

Download history Clear history

Search	Add to builder	Query	Items found	Time
#1	Add	Search (((((("kidney carcinoma") OR ((("renal cell carcinoma" OR "renal cell carcinoma/clear" OR "renal cell carcinoma/clear cell" OR "renal cell carcinoma/tumors")))) OR "renal cell cancer") OR (((kidney cancer[MeSH Terms]) OR cancer of the kidney[MeSH Terms]) OR renal cancer[MeSH Terms]))) AND (((benign tissue[MeSH Terms]) OR normal tissue[MeSH Terms]) OR benign mass[MeSH Terms]))) AND (("biomarkers") OR "immunohistochemistry") Filters: Publication date from 1990/01/01 to 2019/12/31; Humans; English; Middle Aged + Aged: 45+ years; Middle Aged: 45-64 years; Aged: 65+ years; Adult: 19-44 years; Young Adult: 19-24 years; Adult: 19+ years	186	22:56:50

Final search strategy utilized for PubMed database

Antibodies used for immunohistochemistry

Aminopeptidase A / CD 249<br>Invitrogen Catalogue # PA5-27798	Polyclonal unconjugated rabbit IgG.
Aminopeptidase N / CD13<br>Invitrogen Catalogue # MA5-32226.	Recombinant monoclonal unconjugated rabbit IgG
Gamma-glutamyl transferase 1<br>Invitrogen Catalogue # PA5-21344.	Polyclonal unconjugated rabbit IgG.
Neuron-specific enolase<br>Invitrogen Catalogue # MA1-22730.	Monoclonal unconjugated mouse IgG2b.

List of publications included in the systematic review

1. Anastassiou G, Duensing S, Steinhoff G, Zorn U, Grosse J, Dallmann I, et al. Platelet endothelial cell adhesion molecule-1 (PECAM-1): a potential prognostic marker involved in leukocyte infiltration of renal cell carcinoma. Oncology. 1996;53(2):127-32.
2. Baldin AV, Grishina AN, Korolev DO, Kuznetsova EB, Golovastova MO, Kalpinskiy AS, et al. Autoantibody against arrestin-1 as a potential biomarker of renal cell carcinoma. Biochimie. 2019;157:26-37.
3. Bhuvarahamurthy V, Schroeder J, Kristiansen G, Roigas J, Denkert C, Johannsen M, et al. Differential gene expression of urokinase-type plasminogen activator and its receptor in human renal cell carcinoma. Oncology reports. 2005;14(3):777-82.
4. Brasanac D, Müller CA, Müller GA, Hadzi-Dzokic J, Markovic-Lipkovski J. HLA class I antigens expression in renal cell carcinoma: Histopathological and clinical correlation. Journal of Experimental and Clinical Cancer Research. 1999;18(4):505-10.
5. Chang A, Brimo F, Montgomery EA, Epstein JI. Use of PAX8 and GATA3 in diagnosing sarcomatoid renal cell carcinoma and sarcomatoid urothelial carcinoma. Human Pathology. 2013;44(8):1563-1568.
6. Chen W, Ke Z, Shi H, Yang S, Wang L. Overexpression of AEG-1 in renal cell carcinoma and its correlation with tumor nuclear grade and progression. Neoplasma. 2010;57(6):522-9.
7. Cho YE, Lee HL, Lim SJ, Kim KW, Choe BK, Lee S, Park JH. Suppression of GLTSCR2 expression in renal cell carcinomas. Pathology - Research and Practice. 2016;212(2):120-124.
8. Choueiri TK, Fay AP, Gray KP, Callea M, Ho TH, Albiges L, et al. PD-L1 expression in nonclear-cell renal cell carcinoma. Ann Oncol. 2014;25(11):2178-2184.
9. Chun Zhan H, Gudas LJ, Bok D, Rando R, Nanus DM, Tickoo SK. Differential Expression of the Enzyme that Esterifies Retinol, Lecithin: Retinol Acyltransferase, in Subtypes of Human Renal Cancer and Normal Kidney. Clinical Cancer Research. 2003;9(13):4897-905.
10. Ćirović S, Vještica J, Mueller CA, Tatić S, Vasiljević J, Milenković S, et al. NCAM and FGFR1 coexpression and colocalization in renal tumors. International journal of clinical and experimental pathology. 2014;7(4):1402-14.
11. Cochand-Priollet B, Molinié V, Bougaran J, Bouvier R, Dauge-Geffroy MC, Deslignières S, et al. Renal chromophobe cell carcinoma and oncocytoma: A comparative morphologic, histochemical, and immunohistochemical study of 124 cases. Archives of Pathology and Laboratory Medicine. 1997;121(10):1081-6.
12. Demirović A, Džombeta T, Tomas D, Spajić B, Pavić I, Hudolin T, et al. Immunohistochemical expression of tumor antigens MAGE-A3/4 and NY-ESO-1 in renal oncocytoma and chromophobe renal cell carcinoma. Pathology Research and Practice. 2010;206(10):695-9.
13. Demirović A, Ulamec M, Bezjak M, Belicza M, Krušlin B. Apoptotic markers (P53, Bcl-2 and Bax) expression in renal oncocytoma and chromophobe renal cell carcinoma. Periodicum Biologorum. 2014;116(2):173-6.
14. Din NU, Fatima S, Ahmad Z. Chromophobe renal cell carcinoma: A morphologic and immunohistochemical study of 45 cases. Annals of Diagnostic Pathology. 2013;17(6):508-13.
15. Fernández-Aceñero MJ, Cazorla A, Manzarbeitia F. Immunohistochemistry for the differential diagnosis of renal tumors with oncocytic features. Urologic Oncology: Seminars and Original Investigations. 2011;29(5):545-9.
16. Flavin R, Finn SP, Choueiri TK, Ingoldsby H, Ring M, Barrett C, et al. RET protein expression in papillary renal cell carcinoma. Urologic Oncology: Seminars and Original Investigations. 2012;30(6):900-5.
17. Gaut JP, Crimmins DL, Lockwood CM, McQuillan JJ, Ladenson JH. Expression of the Na +/K +-transporting ATPase gamma subunit FXYD2 in renal tumors. Modern Pathology. 2013;26(5):716-24.
18. Geramizadeh B, Ravanshad M, Rahsaz M. Useful markers for differential diagnosis of oncocytoma, chromophobe renal cell carcinoma and conventional renal cell carcinoma. Indian Journal of Pathology and Microbiology. 2008;51(2):167-71.
19. Göhring B, Holzhausen HJ, Meye A, Heynemann H, Rebmann U, Langner J, et al. Endopeptidase 24.11/CD10 is down-regulated in renal cell cancer. International journal of molecular medicine. 1998;2(4):409-14.
20. Golovastova MO, Tsoy LV, Bocharnikova AV, Korolev DO, Gancharova OS, Alekseeva EA, et al. The cancer-retina antigen recoverin as a potential biomarker for renal tumors. Tumor Biology. 2016;37(7):9899-907.
21. Gonzalez-Roibon N, Albadine R, Sharma R, Faraj SF, Illei PB, Argani P, et al. The role of GATA binding protein 3 in the differential diagnosis of collecting duct and upper tract urothelial carcinomas. Hum Pathol. 2013;44(12):2651-7.
22. Guo C, Yang G, Khun K, Kong X, Levy D, Lee P, et al. Activation of Stat3 in renal tumors. American Journal of Translational Research. 2009;1(3):283-90.
23. Ho TH, Kapur P, Joseph RW, Serie DJ, Eckel-Passow JE, Parasramka M, et al. Loss of PBRM1 and BAP1 expression is less common in non-clear cell renal cell carcinoma than in clear cell renal cell carcinoma. Urologic Oncology: Seminars and Original Investigations. 2015;33(1):23.e9-.e14.
24. Högner A, Krause H, Jandrig B, Kasim M, Fuller TF, Schostak M, et al. PBRM1 and VHL expression correlate in human clear cell renal cell carcinoma with differential association with patient's overall survival. Urologic Oncology: Seminars and Original Investigations. 2018;36(3):94.e1-.e14.
25. Horstmann M, Geiger LM, Vogel U, Schmid H, Hennenlotter J, Kuehs U, et al. Kidney-specific cadherin correlates with the ontogenetic origin of renal cell carcinoma subtypes: An indicator of a malignant potential? World journal of urology. 2012;30(4):525-31.

26. Horstmann M, Hennenlotter J, Geiger LM, Vogel U, Schmid H, Kuehs U, et al. Evaluation of the KIT/stem cell factor axis in renal tumours. Anticancer Research. 2012;32(10):4339-46.
27. Ibrahim EC, Allory Y, Commo F, Gattegno B, Callard P, Paul P. Altered pattern of major histocompatibility complex expression in renal carcinoma: Tumor-specific expression of the nonclassical human leukocyte antigen-G molecule is restricted to clear cell carcinoma while up-regulation of other major histocompatibility complex antigens is primarily distributed in all subtypes of renal carcinoma. American Journal of Pathology. 2003;162(2):501-8.
28. Ilhan A, Neziri D, Maj M, Mazal PR, Susani M, Base W, et al. Expression of secretagogin in clear-cell renal cell carcinomas is associated with a high metastasis rate. Human Pathology. 2011;42(5):641-8.
29. Jain S, Roy S, Amin M, Acquafondata M, Yin M, Laframboise W, et al. Amylase α-1A (AMY1A): A novel immunohistochemical marker to differentiate chromophobe renal cell carcinoma from benign oncocytoma. American Journal of Surgical Pathology. 2013;37(12):1824-30.
30. Kim YS, Kim KH, Choi JA, Lee JH, Kim HK, Won NH, et al. Fas (APO-1/CD95) ligand and Fas expression in renal cell carcinomas: correlation with the prognostic factors. Arch Pathol Lab Med. 2000;124(5):687-93.
31. Kraus S, Abel PD, Nachtmann C, Weidner W, Stamp GWH, Chaudhary KS, et al. MUC1 mucin and trefoil factor 1 protein expression in renal cell carcinoma: Correlation with prognosis. Human Pathology. 2002;33(1):60-7.
32. Kruck S, Merseburger AS, Hennenlotter J, Scharpf M, Eyrich C, Amend B, et al. High cytoplasmic expression of p27Kip1 is associated with a worse cancer-specific survival in clear cell renal cell carcinoma. BJU international. 2012;109(10):1565-70.
33. Krüger S, Sotlar K, Kausch I, Horny HP. Expression of KIT (CD117) in renal cell carcinoma and renal oncocytoma. Oncology. 2005;68(2-3):269-75.
34. Kuroda N, Katto K, Tanaka Y, Yamaguchi T, Inoue K, Ohara M, et al. Diagnostic pitfall on the histological spectrum of adult-onset renal carcinoma associated with Xp11.2 translocations/TFE3 gene fusions. Medical Molecular Morphology. 2010;43(2):86-90.
35. Kuroda N, Kanomata N, Yamaguchi T, Imamura Y, Ohe C, Sakaida N, et al. Immunohistochemical application of S100A1 in renal oncocytoma, oncocytic papillary renal cell carcinoma, and two variants of chromophobe renal cell carcinoma. Medical Molecular Morphology. 2011;44(2):111-5.
36. Kuroda N, Inoue K, Guo L, Miyazaki E, Hayashi Y, Naruse K, et al. Expression of CD9/motility-related protein 1 (MRP-1) in renal parenchymal neoplasms: Consistent expression in papillary and chromophobe renal cell carcinomas. Human Pathology. 2001;32(10):1071-7.
37. Kuroda N, Guo L, Toi M, Naruse K, Miyazaki E, Hayashi Y, et al. Paxillin: Application of immunohistochemistry to the diagnosis of chromophobe renal cell carcinoma and oncocytoma. Applied Immunohistochemistry and Molecular Morphology. 2001;9(4):315-8.
38. Langner C, Wegscheider BJ, Ratschek M, Schips L, Zigeuner R. Keratin immunohistochemistry in renal cell carcinoma subtypes and renal oncocytomas: A systematic analysis of 233 tumors. Virchows Archiv. 2004;444(2):127-34.
39. Langner C, Ratschek M, Rehak P, Schips L, Zigeuner R. Expression of MUC1 (EMA) and E-cadherin in renal cell carcinoma: A systematic immunohistochemical analysis of 188 cases. Modern Pathology. 2004;17(2):180-8.
40. Lee HW, Lee EH, Lee CH, Chang HK, Rha SH. Diagnostic utility of caveolin-1 and MOC-31 in distinguishing chromophobe renal cell carcinoma from renal oncocytoma. Korean Journal of Urology. 2011;52(2):96-103.
41. Leroy X, Moukassa D, Copin MC, Saint F, Mazeman E, Gosselin B. Utility of cytokeratin 7 for distinguishing chromophobe renal cell carcinoma from renal oncocytoma. European Urology. 2000;37(4):484-7.
42. Li Q, Zhang XQ, Nie L, Chen GS, Li H, Zhang F, et al. Expression of interferon-γ in human adrenal gland and kidney tumours. British Journal of Cancer. 2007;97(3):420-5.
43. Li M, Wang Y, Song Y, Bu R, Yin B, Fei X, et al. Aberrant dna methyltransferase 1 expression in clear cell renal cell carcinoma development and progression. Chinese Journal of Cancer Research. 2014;26(4):371-81.
44. Liu L, Lan G, Peng L, Xie X, Peng F, Yu S, et al. NDUFA4L2 expression predicts poor prognosis in clear cell renal cell carcinoma patients. Renal Failure. 2016;38(8):1199-205.
45. Liu Z, Fu Q, Lv J, Wang F, Ding K. Prognostic implication of p27Kip1, Skp2 and Cks1 expression in renal cell carcinoma: A tissue microarray study. Journal of Experimental and Clinical Cancer Research. 2008;27(1).
46. Lohi J, Korhonen M, Leivo I, Kangas L, Tani T, Kalluri R, et al. Expression of type IV collagen α1(IV)-α6(IV) polypeptides in normal and developing human kidney and in renal cell carcinomas and oncocytomas. International Journal of Cancer. 1997;72(1):43-9.
47. Macher-Goeppinger S, Aulmann S, Wagener N, Funke B, Tagscherer KE, Haferkamp A, et al. Decoy receptor 3 is a prognostic factor in renal cell cancer. Neoplasia. 2008;10(10):1049-56.
48. Mazal PR, Exner M, Haitel A, Krieger S, Thomson RB, Aronson PS, et al. Expression of kidney-specific cadherin distinguishes chromophobe renal cell carcinoma from renal oncocytoma. Human Pathology. 2005;36(1):22-8.
49. Merseburger AS, Hennenlotter J, Simon P, Ohneseit PA, Kuehs U, Kruck S, et al. Cathepsin D expression in renal cell cancer-clinical implications. European Urology. 2005;48(3):519-26.
50. Mete Ö, Kapran Y, Güllüoglu MG, Kiliçaslan I, Erbil Y, Senyürek YG, et al. Anti-CD10 (56C6) is expressed variably in adrenocortical tumors and cannot be used to discriminate clear cell renal cell carcinomas. Virchows Archiv. 2010;456(5):515-21.

51. Mjønes PG, Nordrum IS, Qvigstad G, Sørdal Ø, Rian LLM, Waldum HL. Expression of erythropoietin and neuroendocrine markers in clear cell renal cell carcinoma. APMIS. 2017;125(3):213-22.
52. Moon A, Lee S. Reduced erg expression in vascular endothelial cells of renal cell carcinoma; a novel marker of tumor progression and poor prognosis. Laboratory Investigation. 2018;98:368-9.
53. Okoń K. Glypican-3 is expressed in chromophobe renal cell carcinomas. Polish Journal of Pathology. 2008;59(1):15-20.
54. Pan CC, Chen PCH, Chiang H. Overexpression of KIT (CD117) in chromophobe renal cell carcinoma and renal oncocytoma. American journal of clinical pathology. 2004;121(6):878-83.
55. Patrício P, Ramalho-Carvalho J, Costa-Pinheiro P, Almeida M, Barros-Silva JD, Vieira J, et al. Deregulation of PAX2 expression in renal cell tumours: Mechanisms and potential use in differential diagnosis. Journal of Cellular and Molecular Medicine. 2013;17(8):1048-58.
56. Patton KT, Tretiakova MS, Yao JL, Papavero V, Huo L, Adley BP, et al. Expression of RON proto-oncogene in renal oncocytoma and chromophobe renal cell carcinoma. American Journal of Surgical Pathology. 2004;28(8):1045-50.
57. Peters I, Rehmet K, Wilke N, Kuczyk MA, Hennenlotter J, Eilers T, et al. RASSF1A promoter methylation and expression analysis in normal and neoplastic kidney indicates a role in early tumorigenesis. Molecular Cancer. 2007;6.
58. Qie GQ, Wang CT, Chu YF, Wang R. Expression of HMGB1/RAGE protein in renal carcinoma and its clinical significance. International journal of clinical and experimental pathology. 2015;8(6):6262-8.
59. Rabien A, Stephan C, Kilic E, Weichert W, Kristiansen G, Miller K, et al. Renal cell neoplasias: Reversion-inducing cysteine-rich protein with Kazal motifs discriminates tumor subtypes, while extracellular matrix metalloproteinase inducer indicates prognosis. Journal of Translational Medicine. 2013;11(1).
60. Rabjerg M, Guerra B, Oliván-Viguera A, Mikkelsen MLN, Köhler R, Issinger OG, et al. Nuclear localization of the CK2α-subunit correlates with poor prognosis in clear cell renal cell carcinoma. Oncotarget. 2017;8(1):1613-27.
61. Raica M, Cimpean AM, Anghel A. Immunohistochemical expression of vascular endothelial growth factor (VEGF) does not correlate with microvessel density in renal cell carcinoma. Neoplasma. 2007;54(4):278-84.
62. Rocca PC, Brunelli M, Gobbo S, Eccher A, Bragantini E, Mina MM, et al. Diagnostic utility of S100A1 expression in renal cell neoplasms: An immunohistochemical and quantitative RT-PCR study. Modern Pathology. 2007;20(7):722-8.
63. Sari A, Calli A, Altinboga AA, Pehlivan FS, Gorgel SN, Balci U, et al. Nucleophosmin expression in renal cell carcinoma and oncocytoma. APMIS. 2012;120(3):187-94.
64. Sarlos DP, Yusenko MV, Peterfi L, Szanto A, Kovacs G. Dual role of KRT17: Development of papillary renal cell tumor and progression of conventional renal cell carcinoma. Journal of Cancer. 2019;10(21):5124-9.
65. Schuetz AN, Yin-Goen Q, Amin MB, Moreno CS, Cohen C, Hornsby CD, et al. Molecular classification of renal tumors by gene expression profiling. Journal of Molecular Diagnostics. 2005;7(2):206-18.
66. Seligson DB, Pantuck AJ, Liu X, Huang Y, Horvath S, Bui MH, et al. Epithelial cell adhesion molecule (KSA) expression: pathobiology and its role as an independent predictor of survival in renal cell carcinoma. Clin Cancer Res. 2004;10(8):2659-69.
67. Skenderi F, Ulamec M, Vranic S, Bilalovic N, Peckova K, Rotterova P, et al. Cystic renal oncocytoma and tubulocystic renal cell carcinoma: Morphologic and immunohistochemical comparative study. Applied Immunohistochemistry and Molecular Morphology. 2016;24(2):112-9.
68. Song W, Huo SW, Lü JJ, Liu Z, Fang XL, Jin XB, et al. Expression of p53 isoforms in renal cell carcinoma. Chinese Medical Journal. 2009;122(8):921-6.
69. Soyupak B, Erdoğan Ş, Ergin M, Seydaoğlu G, Kuzgunbay B, Tansuğ Z. CA9 expression as a prognostic factor in renal clear cell carcinoma. Urologia internationalis. 2005;74(1):68-73.
70. Su Y, Wang W, Xu Y, Liangjun W, Wang Y, Li C, et al. Clinicopathological significance of galectin-1 expression and percentage of galectin-1-expressing T cells in clear-cell renal cell carcinoma. Canadian Urological Association Journal. 2018;12(5):E243-E9.
71. Taki A, Nakatani Y, Misugi K, Yao M, Nagashima Y. Chromophobe renal cell carcinoma: An immunohistochemical study of 21 Japanese cases. Modern Pathology. 1999;12(3):310-7.
72. Tamaskar I, Choueiri TK, Sercia L, Rini B, Bukowski R, Zhou M. Differential expression of caveolin-1 in renal neoplasms. Cancer. 2007;110(4):776-82.
73. Terpe HJ, Störkel S, Zimmer U, Anquez V, Fischer C, Pantel K, et al. Expression of CD44 isoforms in renal cell tumors: Positive correlation to tumor differentiation. American Journal of Pathology. 1996;148(2):453-63.
74. Tong GX, Yu WM, Beaubier NT, Weeden EM, Hamele-Bena D, Mansukhani MM, et al. Expression of PAX8 in normal and neoplastic renal tissues: An immunohistochemical study. Modern Pathology. 2009;22(9):1218-27.
75. Tsimafeyeu I, Khasanova A, Stepanova E, Gordiev M, Khochenkov D, Naumova A, et al. FGFR2 overexpression predicts survival outcome in patients with metastatic papillary renal cell carcinoma. Clinical and Translational Oncology. 2017;19(2):265-8.
76. Valera VA, Li-Ning TE, Walter BA, Roberts DD, Linehan WM, Merino MJ. Protein expression profiling in the spectrum of renal cell carcinomas. Journal of Cancer. 2010;1(1):184-96.
77. Wagener N, Macher-Goeppinger S, Pritsch M, Hüsing J, Hoppe-Seyler K, Schirmacher P, et al. Enhancer of zeste homolog 2 (EZH2) expression is an independent prognostic factor in renal cell carcinoma. BMC Cancer. 2010;10.

78. Wang H, Liu C, Han J, Zhen L, Zhang T, He X, et al. HER2 expression in renal cell carcinoma is rare and negatively correlated with that in normal renal tissue. Oncology Letters. 2012;4(2):194-8.
79. Wang Q, Zhang W, Yang J, Liu YL, Yan ZX, Guo ZJ, et al. High ERα36 expression level and membrane location predict poor prognosis in renal cell carcinoma. Medicine (United States). 2015;94(26).
80. Williamson SR, Eble JN, Cheng L, Grignon DJ. Clear cell papillary renal cell carcinoma: Differential diagnosis and extended immunohistochemical profile. Modern Pathology. 2013;26(5):697-708.
81. Wu SL, Kothari P, Wheeler TM, Reese T, Connelly JH. Cytokeratins 7 and 20 immunoreactivity in chromophobe renal cell carcinomas and renal oncocytomas. Modern Pathology. 2002;15(7):712-7.
82. Yakirevich E, Matoso A, Sabo E, Wang LJ, Tavares R, Meitner P, et al. Expression of the glucocorticoid receptor in renal cell neoplasms: An immunohistochemical and quantitative reverse transcriptase polymerase chain reaction study. Human Pathology. 2011;42(11):1684-92.
83. Yakirevich E, Morris DJ, Tavares R, Meitner PA, Lechpammer M, Noble L, et al. Mineralocorticoid receptor and 11β-hydroxysteroid dehydrogenase type II expression in renal cell neoplasms: A tissue microarray and quantitative RT-PCR study. American Journal of Surgical Pathology. 2008;32(6):874-83.
84. Yasir S, Herrera L, Gomez-Fernandez C, Reis IM, Umar S, Leveillee R, et al. CD10(+) and CK7/RON(-) immunophenotype distinguishes renal cell carcinoma, conventional type with eosinophilic morphology from its mimickers. Applied Immunohistochemistry and Molecular Morphology. 2012;20(5):454-61.
85. Yoo C, Song C, Hong JH, Kim CS, Ahn H. Prognostic significance of perinephric fat infiltration and tumor size in renal cell carcinoma. J Urol. 2008;180(2):486-91; discussion 91.
86. Yu Y, Wang W, Song L, Hu W, Dong C, Pei H, et al. Ecto-5'-nucleotidase expression is associated with the progression of renal cell carcinoma. Oncology Letters. 2015;9(6):2485-94.
87. Yuan J, Liu S, Yu Q, Lin Y, Bi Y, Wang Y, et al. Down-regulation of human leukocyte antigen class I (HLA-I) is associated with poor prognosis in patients with clear cell renal cell carcinoma. Acta Histochem. 2013;115(5):470-4.
88. Zang T, Zhuang L, Zhang Z, Xin D, Guo Y. Expression of beta-catenin in renal cell carcinoma. Chinese medical journal. 2001;114(2):152-4.
89. Zellweger T, Miyake H, July LV, Akbari M, Kiyama S, Gleave ME. Chemosensitization of human renal cell cancer using antisense oligonucleotides targeting the antiapoptotic gene clusterin. Neoplasia. 2001;3(4):360-7.
90. Zhu X, Kanai Y, Saito A, Kondo Y, Hirohashi S. Aberrant expression of beta-catenin and mutation of exon 3 of the beta-catenin gene in renal and urothelial carcinomas. Pathology international. 2000;50(12):945-52.
91. Zimpfer A, Janke S, Hühns M, Schneider B, Kundt G, Zettl H, et al. C-kit overexpression is not associated with KIT gene mutations in chromophobe renal cell carcinoma or renal oncocytoma. Pathology Research and Practice. 2014;210(8):521-5.

Biomarker abbreviations from Table 1

11B-HSD2: 11-beta-hydroxysteroid dehydrogenase 2

AEG-1: Astrocyte Elevated Gene-1

AMACR: Alpha-methylacyl-CoA racemase

CD249: Aminopeptidase A

AMY-1a: Alpha-amylase

BAP1: BRCA1 Associated Protein-1

Bcl-2: B-cell lymphoma 2

CA-IX: Carbonic anhydrase IX

Cav-1: Caveolin-1

CD3: Cluster of Differentiation 3

CD4: Cluster of Differentiation 4

CD8: Cluster of Differentiation 8

CD9: Cluster of Differentiation 9

CD10: Cluster of Differentiation 10

CD11: Cluster of Differentiation 11

APN: Aminopeptidase N

CD13: Cluster of Differentiation 13

CD14: Cluster of Differentiation 14

CD15/LeuM-1: Cluster of Differentiation 15/Lewis M1 antigen

CD26/DP-4: Cluster of Differentiation 26/Dipeptidyl peptidase-4

CD31/PECAM-1: Cluster of Differentiation 31/Platelet Endothelial Cell Adhesion Molecule 1

CD44-6v: Cluster of Differentiation 44 variant 6

CD44-9v: Cluster of Differentiation 44 variant 9

CD44s: Cluster of Differentiation 44 standard

CD56/NCAM: Cluster of Differentiation 56/Neural Cell Adhesion Molecule

CD73: Cluster of Differentiation 73

CD105: Cluster of Differentiation 105

CD117/c-KIT: Cluster of Differentiation 117/c-KIT

CD147/EMMPRIN: Cluster of Differentiation 147/Extracellular Matrix Metalloproteinase Inducer

CK (pan): Cytokeratin (pan)

CK2a: Casein Kinase 2 alpha

CK7: Cytokeratin 7

CK8: Cytokeratin 8

CK8-18L: Cytokeratin 8-18 Like

CK18: Cytokeratin 18

CK19: Cytokeratin 19

CK20: Cytokeratin 20

Cks: Cyclin-dependent kinase subunits
Dcr-3: Decoy receptor 3
DNMT-1: DNA methyltransferase 1
EPCAM: Epithelial Cell Adhesion Molecule
EPO: Erythropoietin
ERa36: Estrogen Receptor alpha 36
Erg: ETS Related Gene
Estrogen Rec.: Estrogen Receptor
EZH2: Enhancer of zeste homolog 2
Fas: Factor Associated Suicide Receptor
FasL: Fas Ligand
FGFR-1: Fibroblast Growth Factor Receptor 1
FGFR-2: Fibroblast Growth Factor Receptor 2
FXYD-2: FXYD domain-containing ion transport regulator 2
GATA-3: GATA Binding Protein 3
GGT: Gamma-Glutamyl Transferase
GLTSCR-2: Glioma Tumor Suppressor Candidate Region Gene 2
GPC-3: Glypican-3
HER-2: Human Epidermal Growth Factor Receptor 2
HLA: Human Leukocyte Antigen
HLA-II: Human Leukocyte Antigen class II
HLA-B,C: Human Leukocyte Antigen B and C
HLA-G: Human Leukocyte Antigen G
HMGB-1: High Mobility Group Box 1
Hsp-27: Heat Shock Protein 27
IFN-γ: Interferon-gamma
KL-1: Kallikrein-1
Ksp-cadherin: Kidney-specific cadherin
LRAT: Lecithin Retinol Acyltransferase
MAGE-A3/4: Melanoma-associated antigen A3/4
MIA: Melanoma Inhibitory Activity
MOC-31: Monoclonal antibody MOC-31
MSH-2: MutS Homolog 2
MUC-1: Mucin 1
N-cadherin: Neuronal cadherin
NDUFA4L2: NADH:Ubiquinone Oxidoreductase Subunit A4 Like 2
NEP: Neprilysin
NPM: Nucleophosmin
NSE: Neuron-specific enolase

NY-ESO-1: New York Esophageal Squamous Cell Carcinoma 1

OSCAR: Osteoclast-associated receptor

p27Kip1: Cyclin-dependent kinase inhibitor 1B

p53: Tumor protein p53

PAX-2: Paired box gene 2

PAX-8: Paired box gene 8

PBMR-1: Para-BenzoQuinone Reductase-like protein

PD-L1: Programmed death-ligand 1

P-Glycoprotein: P-glycoprotein

PSTAT-3: Phosphorylated Signal Transducer and Activator of Transcription 3

RAGE: Receptor for Advanced Glycation End Products

RASSF1A: Ras association domain-containing protein 1

RECK: Reversion-inducing cysteine-rich protein with kazal motifs

RET: Rearranged during Transfection

RON: Recepteur d'Origine Nantais

S100A1: S100 calcium-binding protein A1

SCF: Stem Cell Factor

Skp-2: S-phase kinase-associated protein 2

TFE-3: Transcription Factor E3

TFE-B: Transcription Factor EB

TFF-1 (pS2): Trefoil factor 1

THP: Tamm-Horsfall protein

TPI-1: Triosephosphate isomerase 1

UPA: Urokinase Plasminogen Activator

UPAR: Urokinase Plasminogen Activator Receptor

VEGF: Vascular Endothelial Growth Factor

VHL: Von Hippel-Lindau tumor suppressor

VLA-4: Very Late Antigen-4

Supplementary Figures

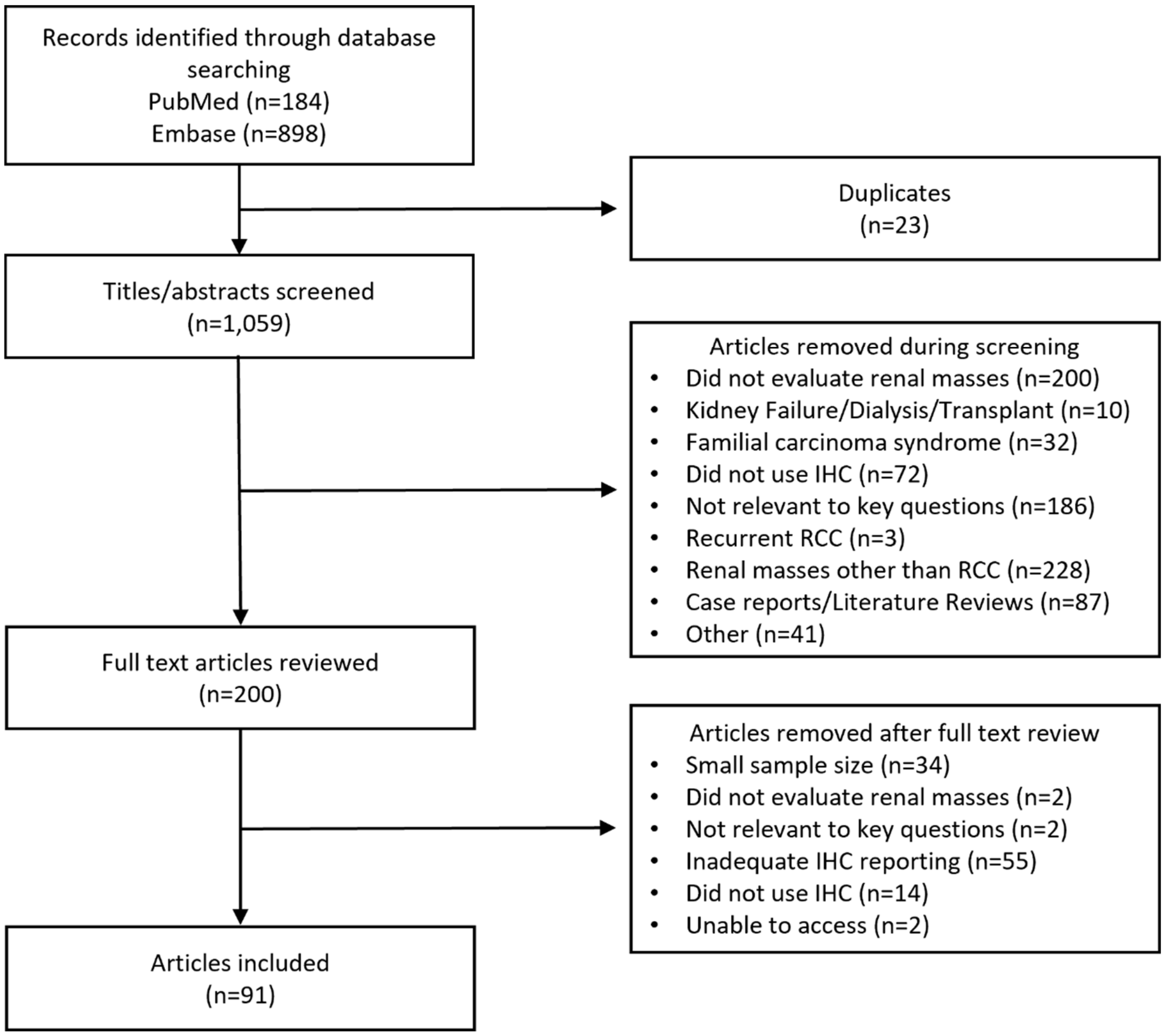

Supplementary Figure 1 – PRISMA diagram for systematic review

Abbreviations: IHC, immunohistochemistry; PRISMA, Preferred Reporting Indices in Systematic Reviews and Meta-analysis; RCC, renal cell carcinoma.

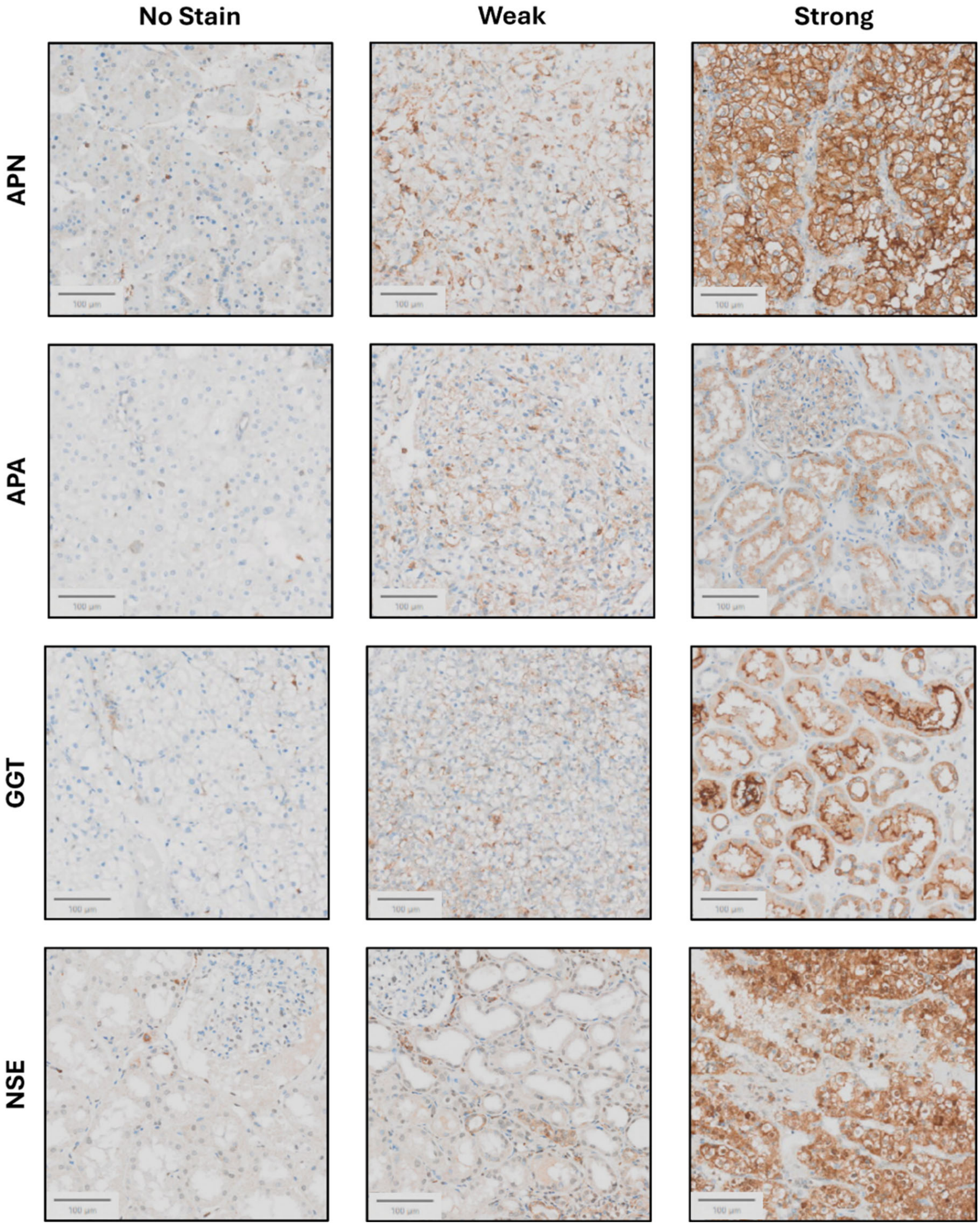

Supplementary Figure 2 – Example images for staining intensity categorization

The images in the figure are representative of the staining intensity of samples assigned to each category. APA, aminopeptidase A; APN, aminopeptidase N; GGT, gamma-glutamyl transferase; NSE, neuron-specific enolase. Scale bar = 100µm.

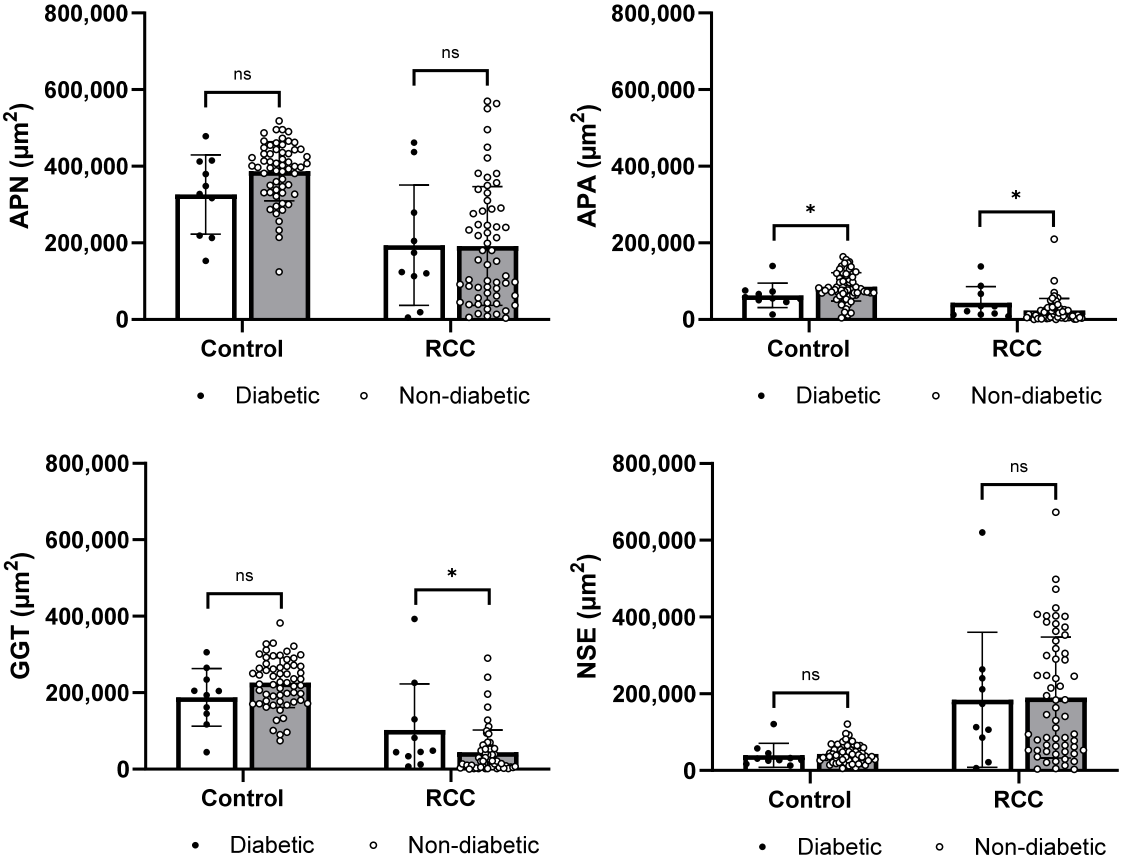

Supplementary Figure 3. Comparison of biomarker detection in diabetic and non-diabetic patients (n=73). Samples were grouped based on diabetic status and total positive staining for each biomarker was compared for both control and RCC tissues. APN and NSE showed no significant difference in positive staining levels in RCC or control (p>0.05). APA showed significantly elevated (p=0.030) positive staining in control tissues and significantly decreased staining (p=0.049) in RCC tissues in non-diabetic patients compared to diabetic patients. GGT showed no significant difference in control tissues (p>0.05), but significantly decreased staining in RCC tissues of non-diabetic patients compared to diabetic (p=0.038). Comparison was performed on GraphPad Prism with Mann-Whitney tests. NS indicates p>0.05, * indicates p<0.05.

Supplementary Tables

**Supplementary Table 1** – Cohort Characteristics			
Demographics	Sample size (n)		73
	Age		32 - 84 (58 avg.)
	Sex		43 male, 30 female
	BMI (kg/M^2)		18.47 - 43.36 (28.7 avg.)
Number of patients with comorbid conditions	Diabetes		10 patients
	HTN		34 patients
	Previous cancer		8 patients
Smoking detail	Previous smokers		23 patients
	Current smokers		10 patients
	Pack years		27 avg. (of those with smoking history)
Biochemistry	Hb		63 - 186 (131.55 avg.)
	MCV		75 - 96 (87.80 avg.)
	WCC		2.2 - 10.9 (7.07 avg.)
	Platelets		97 - 512 (269.44 avg.)
	Urea		3.2 - 23.6 (7.61 avg.)
	Creatinine		53 - 786 (152.84 avg.)
	eGFR		5 - 111 (73.23 avg.)
	CKD stage		1 - 5 (1.95 avg.)
	Albumin		18 - 48 (38.93 avg.)
Tumor details	RCC Grade	1	29
		2	26
		3	11
		4	7
	T	1	44
		2	16
		3	10
		4	3
	N	0	71
		1	2
	M	0	69
		1	4
	Size		11 – 170 cm (68.10 avg.)

**Supplementary Table 2 –** Change in staining intensity					
	Large decrease	Small decrease	No change	Small increase	Large increase
APN	11 (15.1%)	27 (37.0%)	35 (47.9%)	0 (0%)	0 (0%)
APA	6 (8.2%)	49 (67.1%)	18 (24.7%)	0 (0%)	0 (0%)
GGT	12 (16.4%)	50 (68.5%)	11 (15.1%)	0 (0%)	0 (0%)
NSE	0 (0%)	3 (4.1%)	34 (46.6%)	31 (42.5%)	5 (6.8%)

The category of staining intensity for each sample was compared from the control to the RCC tissue samples for each matched pair. Large decrease indicates a sample with strong staining in the control group was then found to have no staining in the RCC group. Small decrease indicates a control sample decreased from strong staining to weak staining, or weak staining to no staining in the matched RCC sample. No change indicates that the matched control and RCC samples had the same category of staining intensity. Large increase indicates a sample with no staining in the control group was then found to have strong staining in the RCC group. Small increase indicates a control sample increased from no staining to weak staining, or weak staining to strong staining in the matched RCC sample.

**Supplementary Table 3. Correlation of total staining values for APN, APA, GGT, and NSE in control tissue samples with various parameters in the patient data (n=73).**				
	APN	APA	GGT	NSE
Age	0.2926	0.4065	0.2937	0.9034
Sex	0.8393	0.4730	0.2038	0.6815
BMI	0.4158	0.2618	0.8439	0.5534
Diabetes	0.0426	0.0851	0.1010	0.6798
HTN	0.5338	0.5141	0.6670	0.0209
Cancer	0.7856	0.4839	0.2084	0.9763
Prev. Smoker	0.8766	0.5657	0.6018	0.1391
Current Smoker	0.6714	0.6543	0.7103	0.6803
Pack years	0.7575	0.2395	0.8319	0.2481
Hb	0.9199	0.4742	0.5546	0.3858
MCV	0.9984	0.1562	0.1234	0.2658
WCC	0.9701	0.8722	0.6750	0.7025
Platelets	0.5657	0.4006	0.1422	0.9525
Urea	0.9496	0.8090	0.8687	0.4254
Creatinine	0.6913	0.8238	0.2241	0.2833
eGFR	0.5111	0.1928	0.5320	0.6629
CKD stage	0.5239	0.1746	0.4350	0.2335
Albumin	0.4337	0.5224	0.2261	0.7508
RCC grade	0.4778	0.0425	0.1846	0.9296
T	0.3056	0.0086	0.2102	0.2421
N	0.8891	0.3861	0.6795	0.4224
M	0.2205	0.3426	0.6451	0.6991
Tumor Size	0.7273	0.0017	0.1542	0.7749

The table shows p-values representing the correlation between the amount of positive staining of each protein in control samples and the indicated patient data parameter. The amount of staining for CD13 was found to have a significant correlation with the prevalence of diabetes ($p=0.0426$) in the selected population sample. The amount of CD249 staining was found to have a significant correlation with RCC grade ($p=0.0425$), T stage ($p=0.0086$), and tumor size (0.0017). The amount of staining of NSE was found to have a significant correlation with the prevalence of hypertension ($p=0.0209$). The amount of staining of GGT1 had no significant correlation with any parameter analyzed ($p>0.05$). Statistical analysis was performed with simple linear regression and simple logistic regression in GraphPad Prism.

**Supplementary Table 4. Correlation of total staining values for APN, APA, GGT, and NSE in RCC tissue samples with various parameters in the patient data (n=73).**				
	APN	APA	GGT	NSE
Age	0.1780	0.5298	0.7472	0.0032
Sex	0.1864	0.2858	0.9600	0.8564
BMI	0.1861	0.0022	0.4229	0.3193
Diabetes	0.9725	0.1217	0.0389	0.9140
HTN	0.9957	0.3891	0.0209	0.5728
Cancer	0.6727	0.9400	0.8930	0.4302
Prev. Smoker	0.7093	0.4999	0.3716	0.9753
Current Smoker	0.3550	0.9220	0.8427	0.4335
Pack years	0.4447	0.7588	0.2521	0.6818
Hb	0.8573	0.3566	0.8072	0.3503
MCV	0.2405	0.3923	0.3907	0.1175
WCC	0.5753	0.2823	0.6207	0.4383
Platelets	0.5021	0.3443	0.4903	0.8850
Urea	0.1216	0.4783	0.7946	0.2797
Creatinine	0.1071	0.2373	0.3711	0.0868
eGFR	0.4501	0.4238	0.8067	0.8390
CKD stage	0.2779	0.3478	0.6732	0.8069
Albumin	0.2729	0.9418	0.8005	0.1649
RCC grade	0.2223	0.7448	0.1072	0.0576
T	0.2035	0.5397	0.2615	0.0077
N	0.1245	0.3491	0.7439	0.1922
M	0.4890	0.6332	0.8405	0.1528
Tumor Size	0.7082	0.3363	0.8890	0.7787

The table shows p-values representing the correlation between the amount of positive staining of each protein in RCC samples and the indicated patient data parameter. The amount of staining for CD249 was found to have a significant correlation with patient BMI ($p=0.0022$). The amount of GGT1 staining was found to have a significant correlation with the prevalence of diabetes ($p=0.0389$) and hypertension ($p=0.0209$). The amount of staining of NSE was found to have a significant correlation with the patient age ($p=0.0032$) and T stage ($p=0.0077$). The amount of staining of CD13 had no significant correlation with any parameter analyzed ($p>0.05$). Statistical analysis was performed with simple linear regression and simple logistic regression in GraphPad Prism.
